# Long-term public antibiotic awareness campaign significantly reduced inappropriate antibiotic use in pediatric primary care settings

**DOI:** 10.3389/fpubh.2026.1730266

**Published:** 2026-02-09

**Authors:** Milica Bajcetic, Dusan M. Spasic, Marija Kukuric, Ivana Lukic, Ana Milijic, Dragana Rajkovic

**Affiliations:** 1 Department of Pharmacology, Clinical Pharmacology and Toxicology, Faculty of Medicine, University of Belgrade, Belgrade, Serbia; 2 Clinical Pharmacology Unit, University Children’s Hospital, Belgrade, Serbia; 3 Department of Emergency Radiology, University Clinical Center of Serbia, Belgrade, Serbia; 4 Project Coordination Unit, Ministry of Health, Republic of Serbia, Belgrade, Serbia; 5 Pharmaceutical Chamber of Serbia, Belgrade, Serbia

**Keywords:** antibiotic awareness, antibiotic consumption, campaign, pediatric, primary care

## Abstract

**Introduction:**

Serbia, like most countries in Southern Europe, has faced persistently high rates of antibiotic consumption. Previous analyses revealed that most antibiotics were prescribed inappropriately, mainly for influenza-like illnesses.

**Methods:**

The first short term media antibiotic awareness campaign (AAC) was held in 2011 and 2014 respectively. Shortly after, in November 2015, the Serbian Ministry of Health launched a nationwide public AAC. The campaign adapted the European Centre for Disease Prevention and Control (ECDC) and World Health Organization (WHO) communication platforms, methodological frameworks, and promotional materials under the motto “Careful with Antibiotics.”

**Results:**

In addition to 423 media features, the campaign encompassed professional education, as well as the development of national guidelines and regulations. Educational activities targeting the public and healthcare professionals (2,119 pediatricians, 428 nurses, and 6,083 pharmacists) were guided by prior analyses of pediatric antibiotic consumption. Over 6 years, 8,750 brochures, 5,500 posters, and more than 3,000 other educational materials were distributed to 158 primary-care centers, pharmacies and in public places nationwide. Long-term campaign interventions resulted in a 32.8% reduction in the number of systemic antibiotic prescriptions per 1,000 children between 2015 and 2021. The most pronounced declines were observed among younger children (2–23 months and 2–11 years respectively) aligning with the main target group of the educational efforts, which focused on young children, including preschool and early school-aged children, who had the highest baseline prescribing rates. Excluding the common cold, the significant decreases were recorded for pharyngitis, tonsillitis, otitis media, and bronchitis, conditions that previously accounted for most inappropriate antibiotic use. From 2011, Serbia’s maximum 61% reduction in pediatric antibiotic prescription rates ranks among the most pronounced in Europe. Despite a gradual shift toward broader-spectrum agents, the Access-group share remained around 60% throughout most of the observation period.

**Conclusion:**

Serbia’s long-term, multisectoral stewardship campaign achieved one of the largest and most sustained declines in pediatric antibiotic consumption across Europe.

## 1 Introduction

Evidence from diverse healthcare systems demonstrates that physician training, parental education, and public awareness campaigns can markedly reduce inappropriate prescribing of antibiotics (1–4). The European Centre for Disease Prevention and Control (ECDC) likewise stresses continuous education, adherence to guidelines, and rigorous surveillance as essential elements of antibiotic stewardship (5, 6). However, short-term interventions often produce only temporary improvements, with prescribing rates returning to baseline after campaigns end (1–3). Recent evaluations across multiple countries including Spain, the United States, Lithuania, Germany, France, Italy, Britain and China, clearly show that ongoing educational and stewardship initiatives can lead to significant and lasting decreases in inappropriate antibiotic prescribing for pediatric patients (7–13).

Antibiotics are among the most frequently prescribed medications in pediatric care, with acute respiratory infections (ARIs) accounting for the majority of prescriptions in primary care (10, 14, 15). An estimated 70%–85% of pediatric antibiotic prescriptions are linked to ARIs, even though most of these infections are viral and self-limiting (14, 15). Inappropriate prescribing provides little clinical benefit while accelerating antimicrobial resistance (AMR), disrupting the developing microbiome, and increasing the risk of adverse drug reactions (14). AMR has been recognized as one of the most pressing public health challenges of the 21st century, contributing to higher morbidity, mortality, and substantial economic costs (16). The Global Burden of Disease collaborators estimated that nearly 5 million deaths in 2019 were associated with AMR, with lower respiratory tract infections contributing the largest share (16). Further analyses highlighted the significant global impact, showing that upper respiratory infections and otitis media are still primary contributors to antibiotic exposure in children, while the overall burden of bacterial AMR is consistently increasing across all regions (10, 14, 16). The detailed datasets reveal a consistent disparity between infection epidemiology and prescribing practices, underscoring the critical need for ongoing international stewardship initiatives.

Beyond mortality, inappropriate antibiotic use drives healthcare expenditures by prolonging hospital stays, increasing reliance on broad-spectrum therapy, and elevating the likelihood of treatment failure (16, 17).

Unfortunately, Serbia exhibits similar patterns to many European countries. Earlier studies documented frequent prescribing for viral infections such as pharyngitis, bronchitis, and otitis media, in direct contradiction to guideline recommendations (18). In order to raise awareness among the community, initial short-term media campaigns were launched by academia in 2011 (19) and 2014 (18) achieving only minor, short-lived reductions. Concurrent pharmacoepidemiological analyses indicated that Serbia exhibited one of the highest antibiotic utilization rates in Europe, driven by widespread over-the-counter access, limited prescription control, and extensive use of broad-spectrum agents (18, 19) These early findings underscored the structural determinants of inappropriate antibiotic use and emphasized the need for a unified, government-driven stewardship strategy rather than fragmented awareness efforts.

Shortly thereafter, in late November 2015, the Ministry of Health of the Republic of Serbia introduced a nationwide campaign as part of the Second Serbia Health Project funded by the World Bank. The campaign under the motto “Careful with Antibiotics” targeted diverse audiences across the healthcare system who directly or indirectly influence the prescription and consumption of antibiotics as well as the community. In addition to media efforts, the campaign included structured education, the production of national guidelines, and regulatory initiatives. Educational activities were tailored to the public (preschool and school children, parents, pregnant women, students, etc.) as well as healthcare professionals (pediatricians, nurses, pharmacists, and others), and were based on analyses of current antibiotic consumption. The media component included public relations activities, press conferences, billboards, and distribution of printed materials. The communication platform, methodological framework, and promotional materials were adapted for Serbian citizens from ECDC and WHO resources. This seven-year national campaign for the rational use of antibiotics was designed to preserve antibiotic effectiveness, reduce antimicrobial resistance, and ultimately lower mortality.

Therefore, to our knowledge, this study is the first to quantify the impact of a long-term, nationwide campaign promoting rational antibiotic use in Serbia’s pediatric population by evaluating decade-long antibiotic consumption quality indicators. In addition, we demonstrate that overall prescribing trends, age-stratified consumption, and diagnosis-specific prescribing patterns can serve as practical metrics for assessing the effectiveness of campaign-style interventions, including media-based and educational initiatives.

## 2 Materials and methods

### 2.1 Research framework and information origins

This study was designed as a retrospective, population-based analysis of pediatric antibiotic prescribing in Serbia from 2011 to 2021, encompassing the period before and after the launch of the national “Careful with Antibiotics” campaign. Prescription data from 2011 to 2018 were obtained from the National Health Insurance Fund (NHIF), which maintains comprehensive records of reimbursed medications issued in state pediatric primary care settings. Beginning in 2019, data collection was conducted through the Integrated Health Information System of Serbia (IHIS), which provides complete documentation of pediatric primary care prescriptions, integrating electronic prescribing functionalities. Both NHIF and IHIS have been established as reliable sources for pharmacoepidemiological studies (18, 20).

The pre-intervention phase (January 2011–November 2015) reflects baseline prescribing patterns prior to the national campaign, whereas the post-intervention phase (December 2015–December 2021) captures the outcomes of ongoing educational, regulatory, and stewardship measures. Annual antibiotic consumption for systemic use (ATC group J01) was quantified as the number of prescriptions per 1,000 children, using population denominators provided by the Statistical Office of the Republic of Serbia. Prescribed antibiotics for systemic use were classified according to the WHO Anatomical Therapeutic Chemical (ATC) system and stratified by both pediatric age group and diagnosis based on the International Classification of Diseases (ICD-10). The study population was divided into four age groups 0–1 month, 2–23 months, 2–11 years, and 12–18 years consistent with established pediatric surveillance standards. Antibiotics were further classified into three groups, Access, Watch and Reserve, according to the WHO AWaRe classification (21).

Indicators for antibiotic consumption and stratification methods were harmonized with the European Surveillance of Antimicrobial Consumption (ESAC) and ECDC quality frameworks, as well as WHO GLASS reporting standards, to ensure international comparability (5, 22). This approach outlines metrics for assessing both the volume and suitability of antibiotic prescriptions. A key aspect is total consumption. The secondary indicators were the distribution of specific antibiotic classes, as well as the equilibrium between narrow- and broad-spectrum agents; Amoxicillin Index, which represents the percentage of phenoxymethylpenicillin and amoxicillin among all pediatric antibiotic prescriptions; and share of at least 60% of antibiotics from the access group according to the WHO AWaRe classification. In addition, we estimated the trend of antibiotic prescriptions for the indications predominantly caused by viral infection: acute pharyngitis (J02), acute tonsillitis (J03), acute bronchitis (J20), common cold (J00), and suppurative otitis media (H65).

### 2.2 Demographics

The study population comprised all children aged 0–18 years enrolled in the Serbian healthcare system. All individuals in the pediatric demographic up to the age of 18 residing in the Republic of Serbia (RS) are entitled to free and mandatory health-insurance coverage. The National Health Insurance Fund (NHIF) provides funding for the network of state-owned health centers, which encompasses the pediatrician-based primary-care system for children. National standards stipulate that children aged 6 years and under treated in pediatric primary-care settings must have access to an ambulance within a 15-min travel distance, while children aged 7 to 18 are cared for by pediatricians integrated within primary-care units associated with schools. During the COVID-19 pandemic, primary pediatric health-care facilities remained accessible to patients.

### 2.3 Overview of the intervention

Since November 2015, Serbia has launched a comprehensive campaign aimed at promoting the rational use of antibiotics. This initiative is coordinated by the Ministry of Health as part of the Second Serbia Health Project, in partnership with professional societies and patient associations. The initiative centered on pediatric care, involving structured educational modules for pediatricians, pharmacists, and other relevant professionals, alongside dissemination of the National Guideline of Good Clinical Practice: The Rational Use of Antibiotics wide-ranging community outreach campaign (23). The outreach work was delivered across television, radio, print, and digital platforms. In addition, the outdoor campaign was designed to reach people in public spaces using visual and concise message delivered via billboards, LED screens, transit ads (bus, trains), posters, stickers etc. Alongside the open-space outreach, posters and other materials were methodically distributed in hallways of primary care facilities.

**Figure 1:**
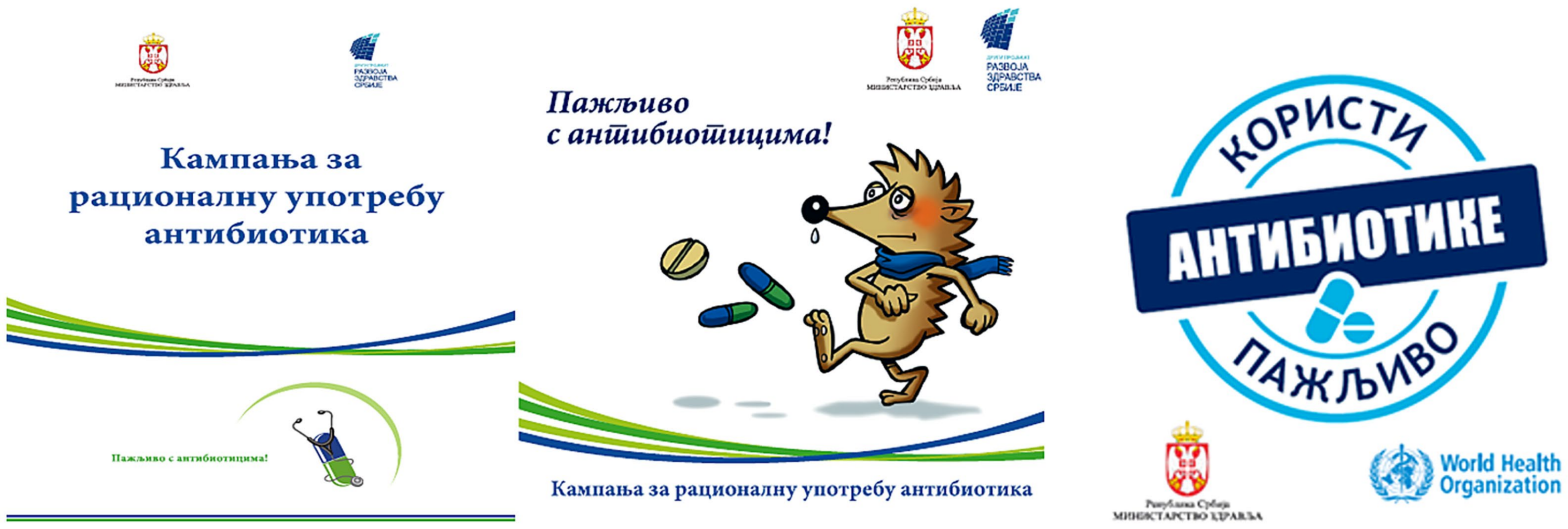
Evolution of the national antibiotic awareness campaign’s visual identity.

The communication platform, methodological framework, and promotional materials were tailored for the Serbian context using resources from ECDC and WHO (24). The visual identity of the campaign, derived from ECDC materials, featured an image of a capsule resembling an antibiotic encircled by a medical stethoscope. The campaign’s motto is “Be Careful with Antibiotics.” Its logo was changed three times during the implementation phases from a capsule wrapped in a stethoscope, and a sniffling hedgehog kicking pills, to a stamp reading “Use Antibiotics Carefully” (Figure 1). The visual identity followed that of the ECDC campaign and later aligned with the WHO initiative. The color palette of green and blue, typically linked to healthcare and medical professionalism, strengthened the campaign’s connection to medicine and public health.

A situational analysis of national prescribing patterns carried out before implementation established the campaign’s behavioral goals: to decrease unnecessary antibiotic prescribing for viral infections, to restrict the use of broad-spectrum agents, to discourage self-medication and over-the-counter use, and to enhance adherence to prescribed therapy. Educational activities were designed to cater to various audiences, encompassing healthcare professionals in pediatric primary care (such as pediatricians, nurses, pharmacists) as well as the general public, including preschool and school children, parents, pregnant women and students (of medicine, pharmacy, veterinary etc). The diagnostic emphasis highlighted conditions associated with documented misuse of antibiotics, especially acute respiratory infections and included recommendations for preventing and addressing therapeutic failure.

The integration of diverse strategies that merge professional education with community involvement has gained international recognition for its effectiveness in enhancing prescribing practices (1, 3, 6). The essential public messages included: Antibiotics are ineffective against colds or flu; Use antibiotics responsibly and only as prescribed by your healthcare provider; and maintaining the effectiveness of antibiotics is a collective responsibility. The campaign’s motto “Careful with Antibiotics” emphasized caution regarding antibiotic use. Supplementary material 1 contains all educational and promotional materials utilized in the campaign.

### 2.4 Statistical analysis

We conducted an analysis of annual pediatric antibiotic prescribing for systemic use covering the period from 2011 to 2021. Each year, the number of prescriptions per 1,000 children was calculated using the corresponding annual pediatric population as the denominator. Descriptive statistics (mean, standard deviation, median, and range) were computed for absolute counts and standardized rates, while year-on-year percentage change and compound annual growth rate (CAGR) were used to describe long-term prescribing dynamics.

Comparisons were made between the pre-intervention period (2011–2015) and the post-intervention period (2016–2021), reflecting the launch of the national campaign in November 2015. Trends over time were analyzed using ordinary least squares (OLS) linear regression to estimate annual changes (slope) with 95% confidence intervals (CI), standardized *β* coefficients, and *p*-values. The interrupted time-series (ITS) framework was applied to assess changes in both level and slope before and after the intervention. Models were fitted with robust standard errors, and autocorrelation was addressed using Prais–Winsten autoregressive [AR (1)] correction. Additional sensitivity analyses were performed excluding 2020–2021 to control for possible confounding related to the COVID-19 pandemic.

All statistical analyses were carried out in EZR (Easy R, version 1.61), while advanced computations, including estimation of the Herfindahl–Hirschman Index (HHI) for prescribing diversity, bootstrap-derived confidence intervals, and data visualization, were implemented using custom scripts in Python 3.11 with the pandas, statsmodels, and matplotlib libraries. This dual-platform workflow ensured transparent cross-validation of results and facilitated the reorganization of figures and tables.

All tests were two-sided, with statistical significance defined as *p* < 0.05. Figures indicate the start of the national campaign (November 2015) and the onset of the COVID-19 pandemic (2020), with shaded areas denoting pre-campaign and campaign periods.

### 2.5 Ethical considerations

The study used aggregated, anonymized national-level data, with no individual patient identifiers. In accordance with national regulations, formal ethical approval was not required. The analysis adhered to the principles of the Declaration of Helsinki.

#### 2.5.1 Limitations

This study has several limitations that should be acknowledged. First, it included only state-owned pediatric primary care facilities covered by the National Health Insurance Fund. Pediatric services in Serbia are also provided by private clinics and individual practices, but data from these settings were unavailable. Although private-sector healthcare professionals participated in national educational and stewardship programs, antibiotic consumption data from private clinics were not captured. Future studies should integrate both public and private datasets to enable a more comprehensive national evaluation of antibiotic use.

The ecological study design restricts the ability to infer direct causality between the educational campaign and the observed decline in antibiotic use. While interrupted time-series modeling strengthened causal inference by accounting for pre-existing trends, other contextual influences, such as concurrent policy changes, variations in drug market availability, or socioeconomic shifts, may also have contributed to the results.

The analysis focused on prescription counts rather than doses or duration, preventing assessment of total antibiotic exposure. Likewise, inpatient prescribing, self-medication, and private over-the-counter antibiotic use were not captured, which could lead to a slight underestimation of actual community consumption.

Finally, the COVID-19 pandemic introduced disruptions in healthcare utilization and disease incidence that may have temporarily influenced prescribing patterns independently of stewardship interventions.

## 3 Results

### 3.1 Summary of executed strategies

Over the course of 6 years, the national campaign implemented a series of coordinated interventions encompassing media outreach, professional and community education, and the development of key policy documents. These initiatives were aligned with the WHO Global Action Plan on Antimicrobial Resistance, adopted on March 27, 2015. Within this framework, Serbia introduced the National Antibiotic Resistance Control Programme and issued the National Guideline of Good Clinical Practice: Rational Use of Antibiotics (25). Since 2015, November 18 has been officially recognized as the European Day for the Rational Use of Antibiotics, marking the start of the World Antibiotic Awareness Week in Serbia. In 2019, an additional qualitative step forward was achieved through the establishment of a National multisectoral coordination group on antimicrobial resistance control that, in addition to human medicine, also encompassed veterinary, agricultural, and environmental sectors - One Health approach, thereby strengthening coordination in the national response to antimicrobial resistance.

### 3.2 Media initiative

The National Media Campaign for the Rational Use of Antibiotics comprised 423 media features, including 82 national and 21 local television reports, 12 radio broadcasts, 98 national and 15 local print articles and 195 online media releases.

Educational and promotional materials were extensively disseminated, comprising 8,750 brochures for the general public and multiple fact sheets tailored to healthcare professionals: 1,750 for primary care prescribers, 1,250 to support physician–patient communication, 420 for secondary care prescribers, and 850 each on antibiotic consumption and resistance in the European Union. In addition, 2,500 posters and two video spots were developed and distributed nationwide.

Public engagement activities, including press conferences, community events, and public forums were supported by branded campaign materials (1,720 bags, 600 cups, 100 T-shirts, 50 scarves, 4 roll-up banners, and one life-size campaign mascot).

A large-scale advertising effort targeted both the general population and healthcare professionals in primary care. Three thousand posters were distributed to 158 health centers and to state and private pharmacies across the country. The visual campaign also included over 30 illuminated billboards, 45 double-sided light panels on public poles, and displays placed in 25 buses and at 13 railway stations, ensuring high visibility in urban areas with heavy pedestrian traffic.

### 3.3 Learning initiatives

Targeted educational programs were conducted within pediatric and primary care settings. A total of 2,119 pediatricians and other pediatric specialists participated in structured training sessions designed to promote the rational use of antibiotics. Additionally, 428 pediatric nurses from preschool institutions, along with 715 parents and preschool children, took part in tailored workshops addressing antibiotic misuse and infection prevention.

During the final 2 years of the campaign, 6,083 pharmacists completed accredited online training programs on antimicrobial stewardship and rational prescribing practices.

**Figure 2:**
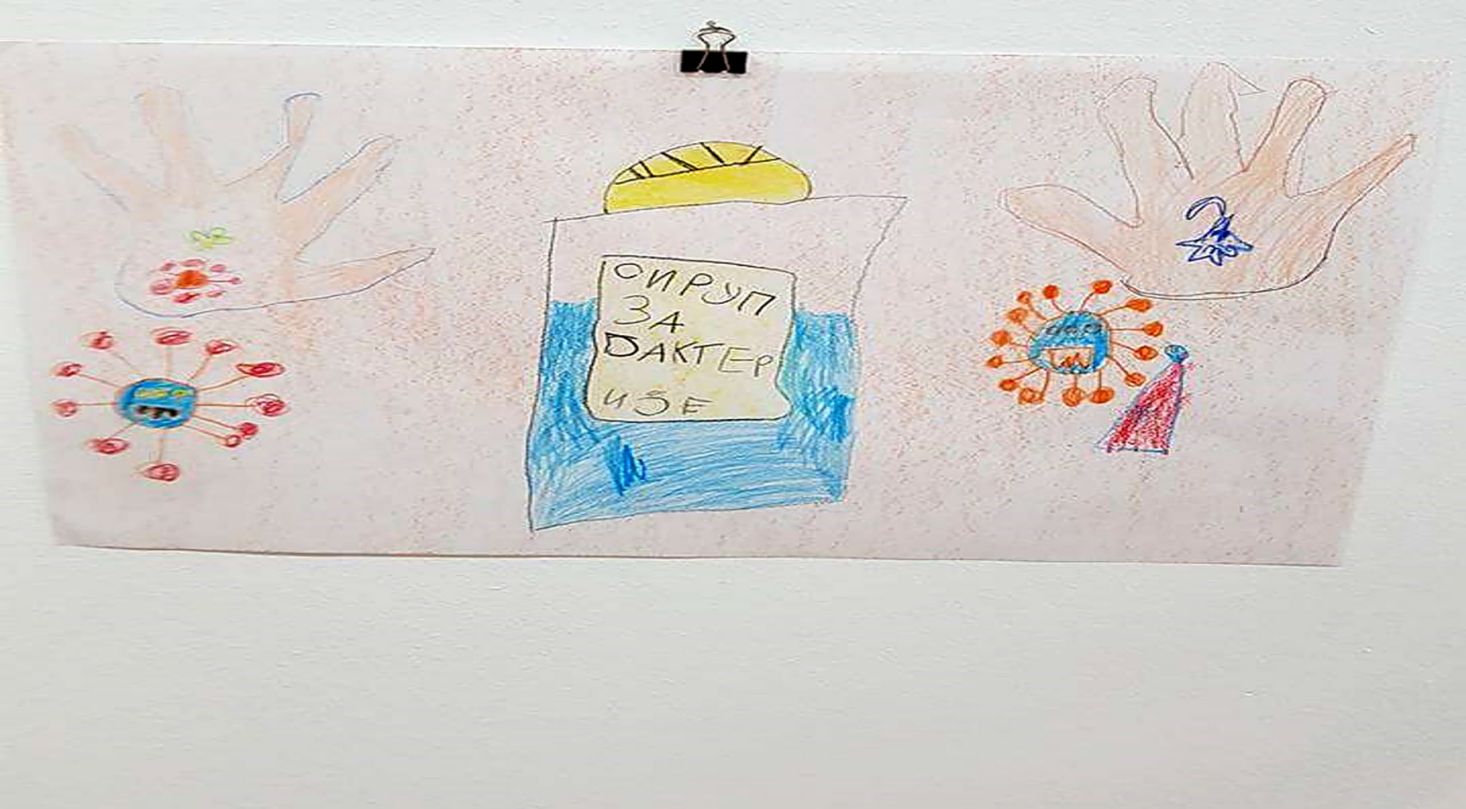
Artwork in preschool institutions on the topic of “Antibiotics and bacteria”. The label on the bottle reads: “Syrup for bacteria”.

In collaboration with professional associations, NGOs, and local authorities, a series of community-based educational initiatives were implemented across Serbia’s largest cities Belgrade, Novi Sad, Niš, and Kragujevac. Four public forums titled “Rational Use of Antibiotics: A Shared Responsibility” gathered 360 participants, complemented by additional community forums held during the Spring and Autumn Health Festivals in Belgrade. Art competitions on the theme “Antibiotics and Bacteria” engaged 700 preschool children, with 160 awardees (Figure 2), while workshops for expectant parents on the rational use of antibiotics involved 395 participants.

**Figure 3:**
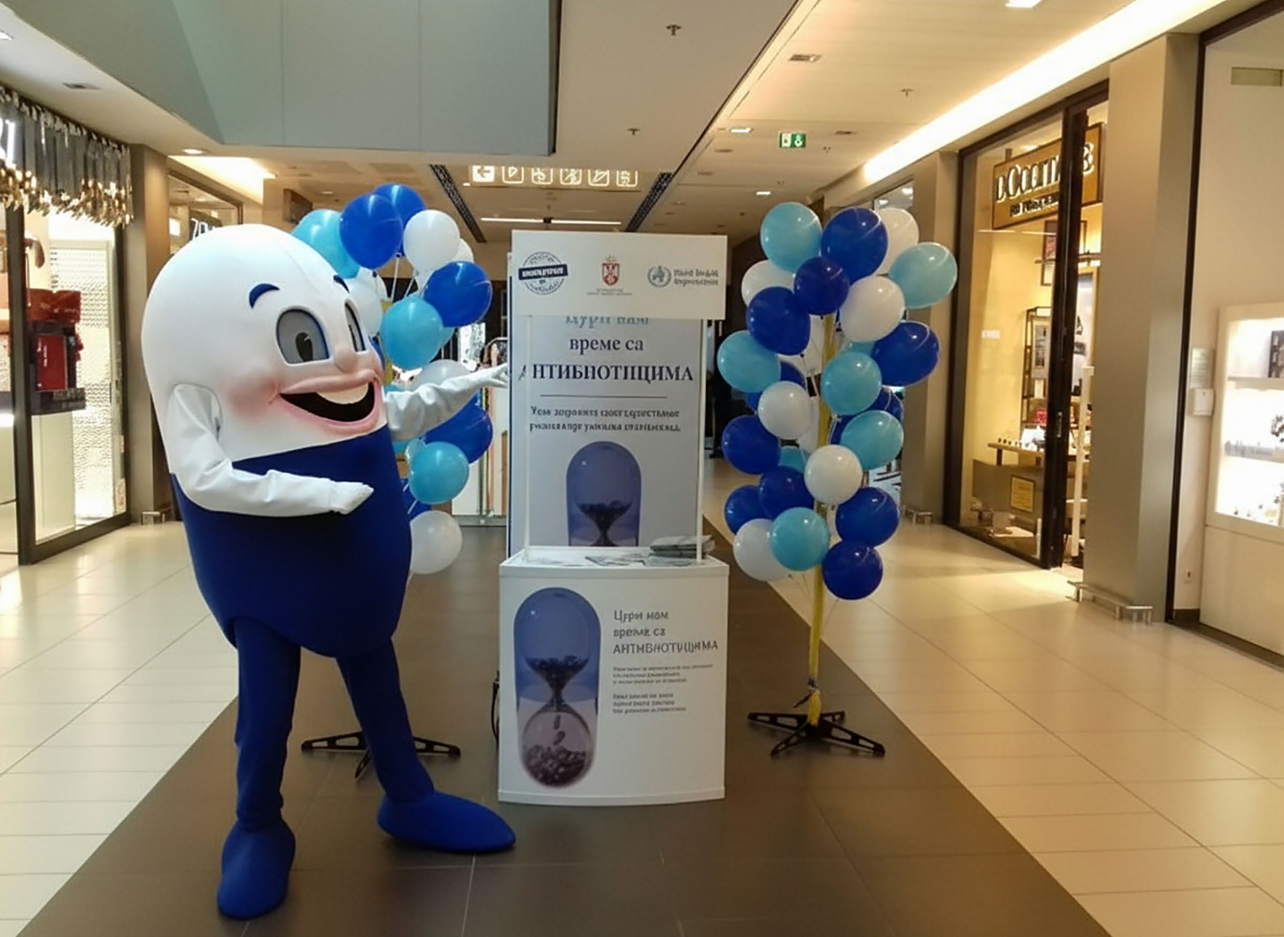
The World Awareness Week on the Rational Use of Antibiotics (18–24 November).

Additional cooperation with medical and pharmacy students led to community outreach activities that were organized in busy urban areas and shopping centers across Belgrade, Novi Sad, Nis, and Kragujevac (Figure 3).

### 3.4 Total antibiotic prescriptions

#### 3.4.1 Descriptive analysis

Between 2011 and 2021, the total number of pediatric antibiotic prescriptions declined markedly, from 2,072,519 to 1,167,381 an absolute reduction of 905,138 prescriptions (−43.7%). The annual mean across the decade was 1,583,095 (±366,933 SD; median 1,707,577). From 2011 to 2017, prescribing remained relatively stable, ranging between approximately 1.6 and 2.0 million prescriptions per year. A pronounced decline began in 2018, culminating in a sharp 33.7% year-on-year decrease in 2020, largely attributable to the COVID-19 pandemic. A partial rebound followed in 2021 (+41.7%), though totals remained well below pre-pandemic levels.

### 3.5 Number of prescriptions per 1,000 children

#### 3.5.1 Descriptive analysis

**Figure 4:**
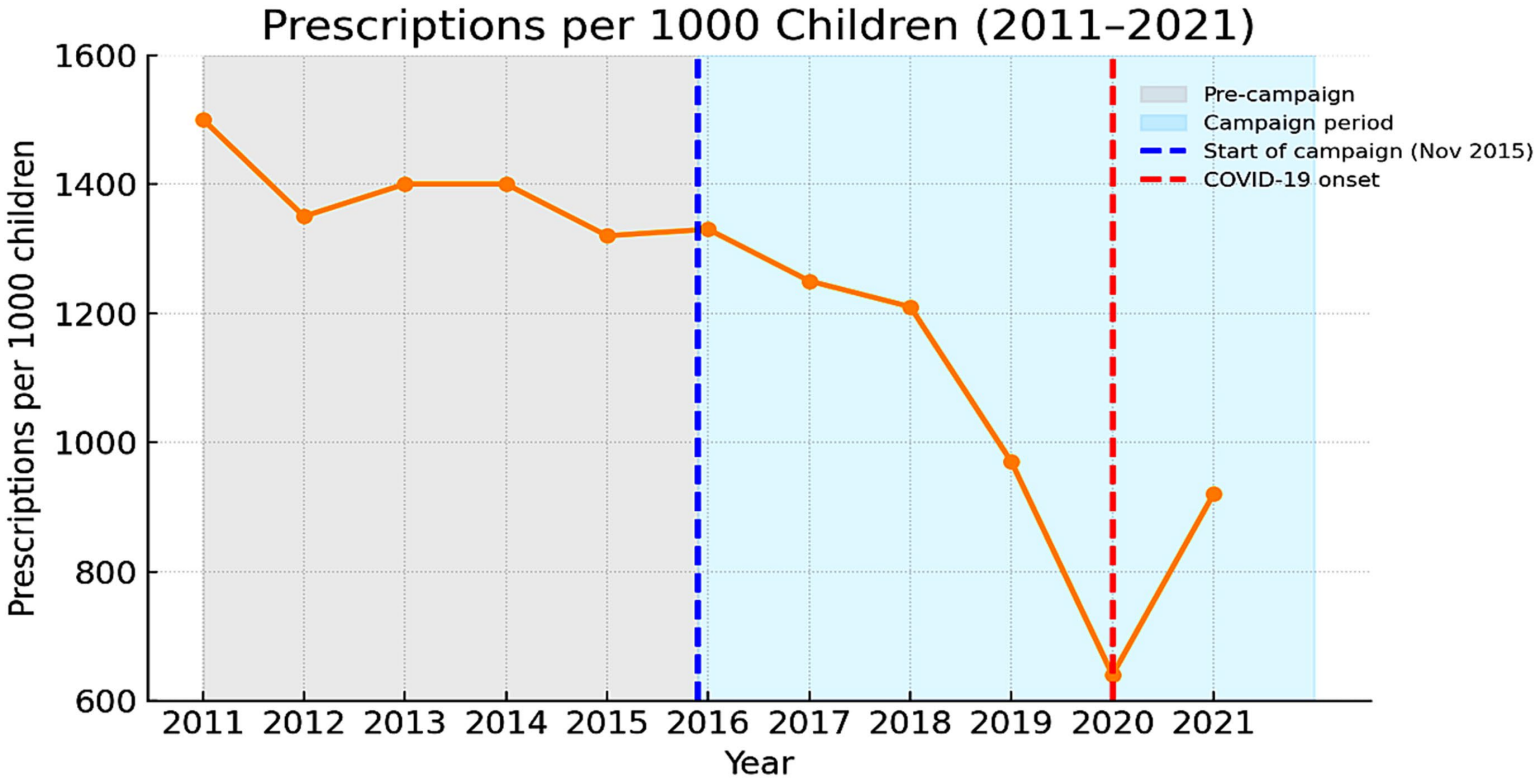
Rate of pediatric antibiotic prescriptions for systemic use from 2011 to 2021.

**Figure 5:**
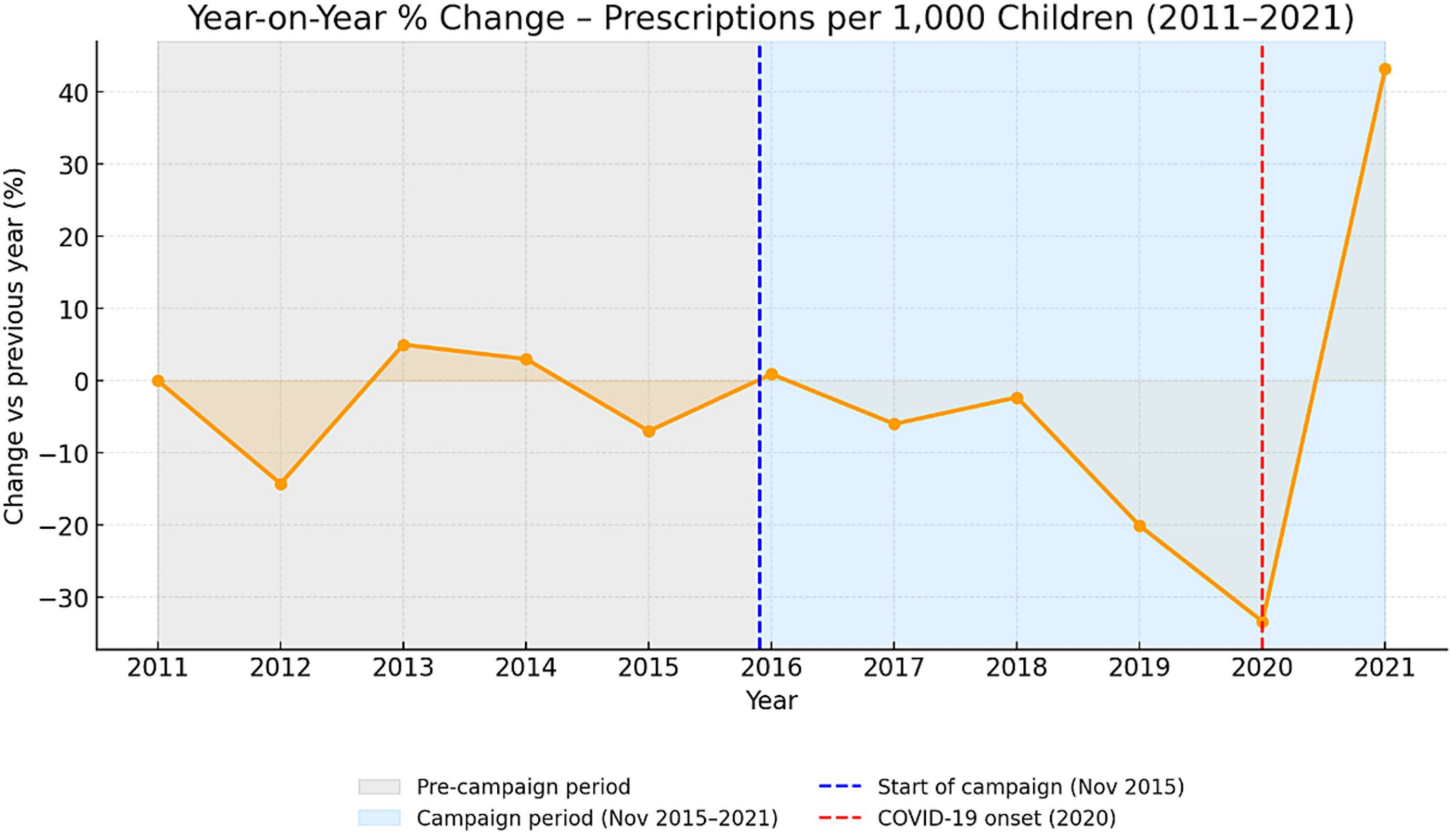
Year-on-year percentage change in number of prescriptions per 1,000 children, 2011–2021.

Standardizing by the pediatric population produced similar results. Prescription density decreased from 1,516 per 1,000 children in 2011 to 927 in 2021 an absolute reduction of 589 per 1,000 (−38.9%). The mean rate was 1,208 (±257 SD; median 1,308) prescriptions per 1,000 children per year. While the normalized trend appeared smoother than the absolute totals, it followed the same pattern a gradual pre-2016 decline, a sharp decrease in 2020, and partial recovery in 2021 (Figure 4). The year-on-year percentage changes in prescription density are presented in Figure 5. Modest annual fluctuations were observed before the 2016 campaign, followed by a sequence of steeper declines during 2017–2020, culminating in a marked contraction at the onset of the COVID-19 pandemic. A partial rebound occurred in 2021, though overall prescribing rates remained well below pre-campaign levels. These dynamics confirm that the long-term decrease in pediatric antibiotic use was gradual and sustained, with pandemic-related disruptions further amplifying the downward trajectory.

#### 3.5.2 Interrupted time-series regression (segmented OLS with robust SEs)

Before 2016, prescription rates declined by −34.38 per 1,000 children annually (*p* = 0.076). No significant immediate change was recorded at the campaign onset (+526.39; *p* = 0.065). After 2016, the slope steepened to −79.56 per 1,000 children per year (*p* = 0.064; R^2^ = 0.839). Although marginally significant, the post-campaign trend indicates an intensified and progressive decline consistent with a cumulative educational effect.

#### 3.5.3 Autocorrelation-adjusted regression (Prais–Winsten, AR (1))

After adjusting for serial correlation, the pre-2016 decline was reduced and rendered non-significant (−16.98 per 1,000 children per year; *p* = 0.638). The immediate level change displayed a minor positive shift (+680.10; *p* = 0.018), while the post-2016 slope change achieved statistical significance (−108.37 per 1,000 children per year; *p* = 0.030). These findings indicate a long-term acceleration of decline after 2016, once again illustrating gradual adaptation in prescribing behavior rather than a sudden transition. In the Prais–Winsten AR (1) model, the campaign period was associated with a 32.8% reduction in pediatric antibiotic prescription rates relative to the counterfactual trend.

#### 3.5.4 Mean difference and effect size

The mean annual prescribing density decreased from 1,392.65 to 1,053.67 per 1,000 children (*t* = −3.10, *p* = 0.020). The effect size was large (Cohen’s d = −1.73; Hedges’ *g* = −1.58), representing an average reduction of approximately 339 prescriptions per 1,000 children each year.

#### 3.5.5 Percentage change with 95% confidence interval

The standardized prescribing rate decreased by −24.3% (95% CI −38.5 to −11.1%). Together with the absolute results, these findings provide robust statistical evidence of a long-term and sustained reduction in pediatric antibiotic use during and after the national campaign period.

### 3.6 Age-specific trends in pediatric antibiotic prescribing

#### 3.6.1 Descriptive analysis

**Figure 6:**
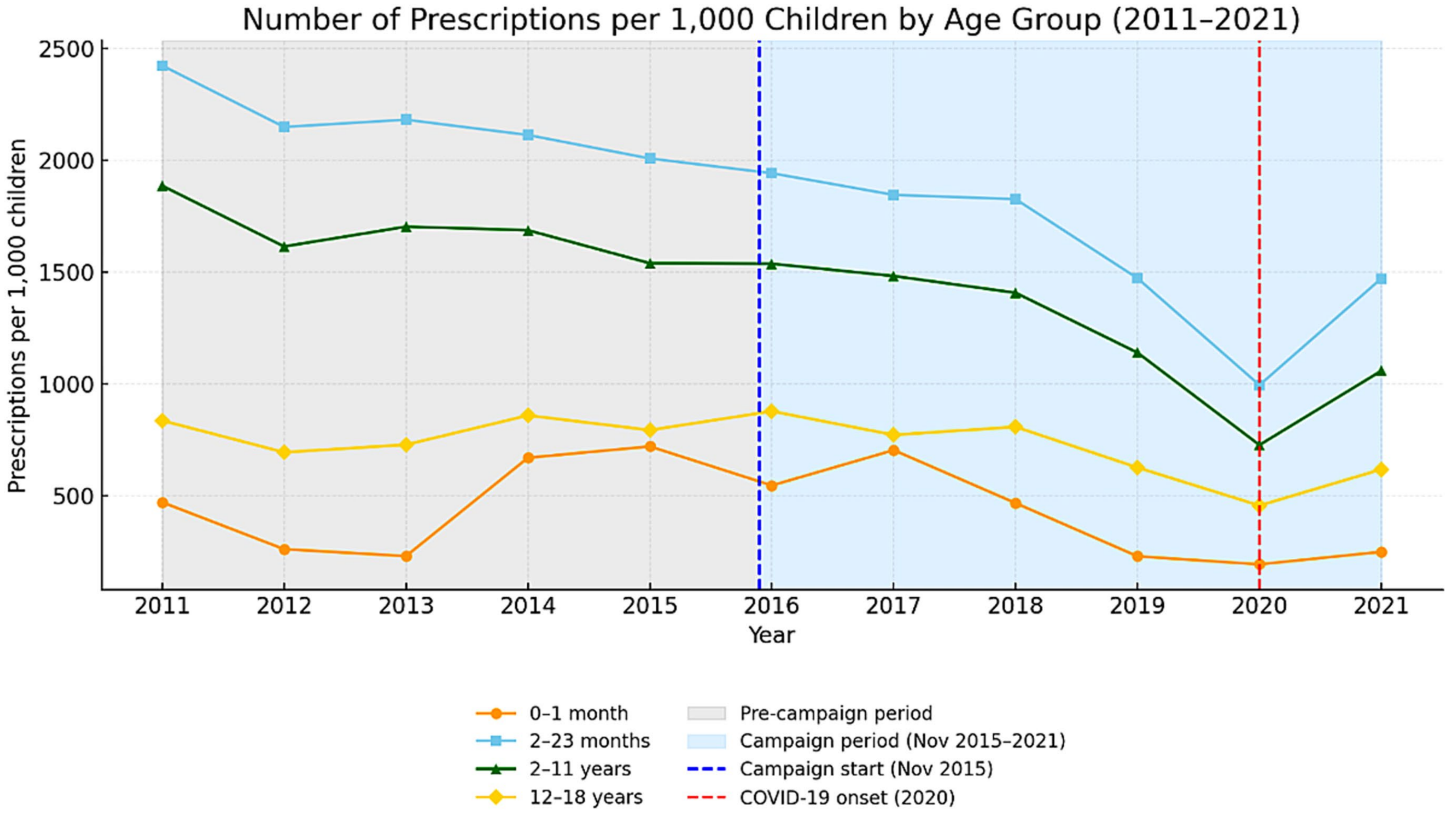
Antibiotic prescription rates by age group (2011–2021). [The blue and red dashed lines mark the start of the educational campaign (November 2015) and the onset of COVID-19 (2020). Gray and blue shading indicate pre-campaign and campaign periods.].

From 2011 to 2021, there was notable variability in antibiotic prescribing across pediatric age groups, with the most substantial reductions observed among younger children (Figure 6). Children aged 2–23 months and 2–11 years exhibited the highest consumption levels, both showing consistent long-term declines. In contrast, prescription rates for newborns (0–1 month) and adolescents (12–18 years) remained relatively stable throughout the decade.

Among infants aged 2–23 months, the average prescribing rate declined from approximately 2,217 to 1,640 prescriptions per 1,000 children (−26%). Similarly, for children aged 2–11 years, there was a comparable decrease from 1,722 to 1,295 per 1,000 (−25%). Adolescents showed only a modest 8% reduction, while prescribing among neonates remained constant at around 400 prescriptions per 1,000 children. The lowest values were observed in 2020, coinciding with the COVID-19 pandemic, followed by a partial rebound in 2021. Overall, the most significant and lasting improvements were observed among younger children, who historically exhibited the highest rates of antibiotic overuse.

### 3.7 Linear trend modeling

Linear regression analysis revealed statistically significant downward trends in the two younger cohorts. Among children aged 2–23 months, there was an average decline of 97 prescriptions per 1,000 children annually (*p* < 0.001; *R*^2^ = 0.78), representing the strongest reduction across all subgroups. Likewise, children aged 2–11 years exhibited an average annual decrease of 68 prescriptions per 1,000 children (*p* = 0.003; *R*^2^ = 0.59). In the neonatal group (0–1 month), there was a non-significant reduction of 23 prescriptions per 1,000 per year (*p* = 0.21; *R*^2^ = 0.15), while adolescents (12–18 years) demonstrated a small, non-significant decline of 16 prescriptions per 1,000 children per year (*p* = 0.13; *R*^2^ = 0.21). These findings suggest that the most substantial and statistically supported progress occurred among younger pediatric populations.

### 3.8 Interrupted time-series analysis by subgroup

Segmented regression analysis indicated no statistically significant immediate effects of the 2015 national educational campaign in any specific age group. Nonetheless, overall patterns reflected a gradual and sustained change in prescribing behavior rather than an abrupt shift. A potential downward inflection was identified among neonates after 2015, with the slope decreasing by 142 prescriptions per 1,000 per year (*p* ≈ 0.08). Among infants aged 2–23 months and children aged 2–11 years, the downward trend persisted throughout both the pre- and post-campaign periods, with no significant “step change” at 2015 (*p* > 0.80). In adolescents, the model demonstrated minimal variation and low explanatory power (*p* > 0.40). These results suggest that the campaign’s effect was cumulative rather than instantaneous.

### 3.9 Trends in antibiotic consumption by pharmacological group (2011–2021)

The overall volume of antibiotic prescriptions per 1,000 children displayed clear variations among different antibiotic classes throughout the study period (Tables 1, 2). From 2011 to 2015, penicillins were the most commonly prescribed class, with an average of 497 prescriptions per 1,000 children, followed by macrolides (268 per 1,000) and first-generation cephalosporins (277 per 1,000). In the pre-intervention phase (Table 1), penicillin use exhibited a slight, non-significant upward trend (CAGR = +8.13%, *β* = 0.58, *p* = 0.303), whereas prescriptions for β-lactamase inhibitor combinations showed a minor decline (CAGR = −10.28%, *β* = −0.81, *p* = 0.098). Both first- and second-generation cephalosporins demonstrated moderate decreases (CAGR = −6.82% and −29.72%, respectively), indicating a gradual replacement by newer oral cephalosporins. The use of macrolides declined modestly (CAGR = −8.02%), while prescriptions for fluoroquinolones though infrequent in absolute numbers showed a small yet statistically significant increase (*p* = 0.005). The ratio of broad to narrow-spectrum agents (J01_B/N) exhibited a consistent downward trend during this period, with a compound annual reduction of −11.1%, reflecting improved preference for narrow-spectrum agents in the pre-intervention years.

**Table 1:** Number of prescriptions per 1,000 children for antibiotic classes during the pre-intervention period (2011–2015).^a^

Class^b^	2011	2012	2013	2014	2015	CAGR^c^ (%)	B^c^	*β*^c^	*p*^c^
Penicillins	362.80	516.59	566.59	544.17	496.03	8.13	29.40	0.58	0.303
Pen + β-lactamase inhibitor	309.87	221.38	206.14	201.27	200.83	−10.28	−23.82	−0.81	0.098
Ceph 1st gen	329.21	259.15	279.80	268.96	248.22	−6.82	−15.22	−0.77	0.131
Ceph 2nd gen	80.54	9.89	17.98	18.76	19.65	−29.72	−11.29	−0.62	0.266
Ceph 3rd gen	61.95	40.03	46.26	69.26	69.69	2.99	4.47	0.52	0.369
Macrolides	336.01	251.29	246.60	267.89	240.50	−8.02	−17.44	−0.71	0.183
Fluoroquinolones	1.47	2.47	2.63	3.25	3.61	25.23	0.51	0.97	0.005
J01_B/N^d^	0.64	0.35	0.32	0.36	0.40	−11.1%	/	/	/

a Yearly prescription rates are normalized by pediatric population size.

b Values represent aggregated totals for each pharmacological group.

c CAGR, compound annual growth rate; *β*, standardized regression coefficient; *p*, statistical significance.

d The ratio of consumption of mainly “broad-spectrum” penicillins, “broad-spectrum” cephalosporins, macrolides (except erythromycin) and fluoroquinolones [ATC groups J01(CR+DC+DD+(FA– FA01) + MA)] to the consumption of narrow-spectrum penicillins, narrow-spectrum cephalosporins and erythromycin [ATC groups J01(CA+CE+CF+DB+FA01)].

**Table 2:** Number of prescriptions per 1,000 children for antibiotic classes during the post-intervention period (2016–2021).^a^

Class	2016	2017	2018	2019	2020	2021	CAGR^b^ (%)	B^b^	*β*^b^	*p*^b^
Penicillins	477.91	420.80	383.15	287.01	173.47	237.47	−13.05	−58.30	−0.94	0.006
Pen + β-lactamase inhibitor	212.40	211.69	229.63	208.29	123.98	185.03	−2.72	−12.04	−0.60	0.210
Ceph 1st gen	234.27	216.20	191.70	136.58	86.92	94.05	−16.68	−32.69	−0.97	0.001
Ceph 2nd gen	21.83	28.04	22.56	25.24	17.99	37.46	11.41	1.45	0.40	0.430
Ceph 3rd gen	87.78	113.66	130.99	120.82	90.58	163.93	13.31	8.61	0.57	0.236
Macrolides	256.23	221.73	227.59	173.73	139.96	189.27	−5.88	−18.11	−0.81	0.052
Fluoroquinolones	3.01	3.16	3.28	1.70	1.90	2.15	−6.46	−0.28	−0.74	0.092
J01_B/N^c^	0.47	0.58	0.69	0.84	0.90	1.16	19.81	/	/	/

a Data reflect population-adjusted prescription rates across principal antibiotic groups following the onset of national educational interventions.

b CAGR, compound annual growth rate; β, standardized regression coefficient; *p*, statistical significance.

c The ratio of consumption of mainly “broad-spectrum” penicillins, “broad-spectrum” cephalosporins, macrolides (except erythromycin) and fluoroquinolones [ATC groups J01(CR+DC+DD+(FA– FA01) + MA)] to the consumption of narrow-spectrum penicillins, narrow-spectrum cephalosporins and erythromycin [ATC groups J01(CA+CE+CF+DB+FA01)].

During the period from 2016 to 2021 (Table 2), overall pediatric antibiotic prescribing declined significantly across all classes, accompanied by notable qualitative differences. Penicillins showed the sharpest decline, with a compound annual growth rate of −13.05% (*β* = −0.94, *p* = 0.006), decreasing from approximately 478 to 237 prescriptions per 1,000 children. A substantial reduction was also observed in first-generation cephalosporins (CAGR = −16.68%, *p* = 0.001). In contrast, third-generation cephalosporins, including cefixime, demonstrated an increase in relative share (CAGR = +13.31%), reflecting a gradual shift toward broader-spectrum coverage. Combinations of β-lactamase inhibitors, particularly amoxicillin–clavulanate, exhibited a slight, non-significant decrease (CAGR = −2.72%, *p* = 0.210), indicating their persistent clinical prominence despite stewardship initiatives. In contrast, the J01_B/N ratio rose sharply after 2016, with a compound annual growth rate of +19.81%, indicating a marked post-intervention shift toward broader-spectrum agent use.

Macrolide consumption declined steadily following the intervention (CAGR = −5.88%, *p* = 0.052), with azithromycin remaining the most frequently prescribed agent. This pattern is consistent with previous national findings suggesting continued macrolide overuse in pediatric respiratory infections. Fluoroquinolone prescribing remained very low (<3 prescriptions per 1,000 children) and showed no significant variation, aside from a brief rise observed during 2018–2019.

In essence, although overall antibiotic use among children declined, the distribution across classes revealed a shift toward broader-spectrum agents particularly third-generation cephalosporins and amoxicillin-clavulanate (Table 2). These findings suggest that stewardship interventions effectively reduced total prescribing volume but were less successful in optimizing spectrum selection.

### 3.10 Prescribing diversity (Herfindahl–Hirschman index, HHI)

**Figure 7:**
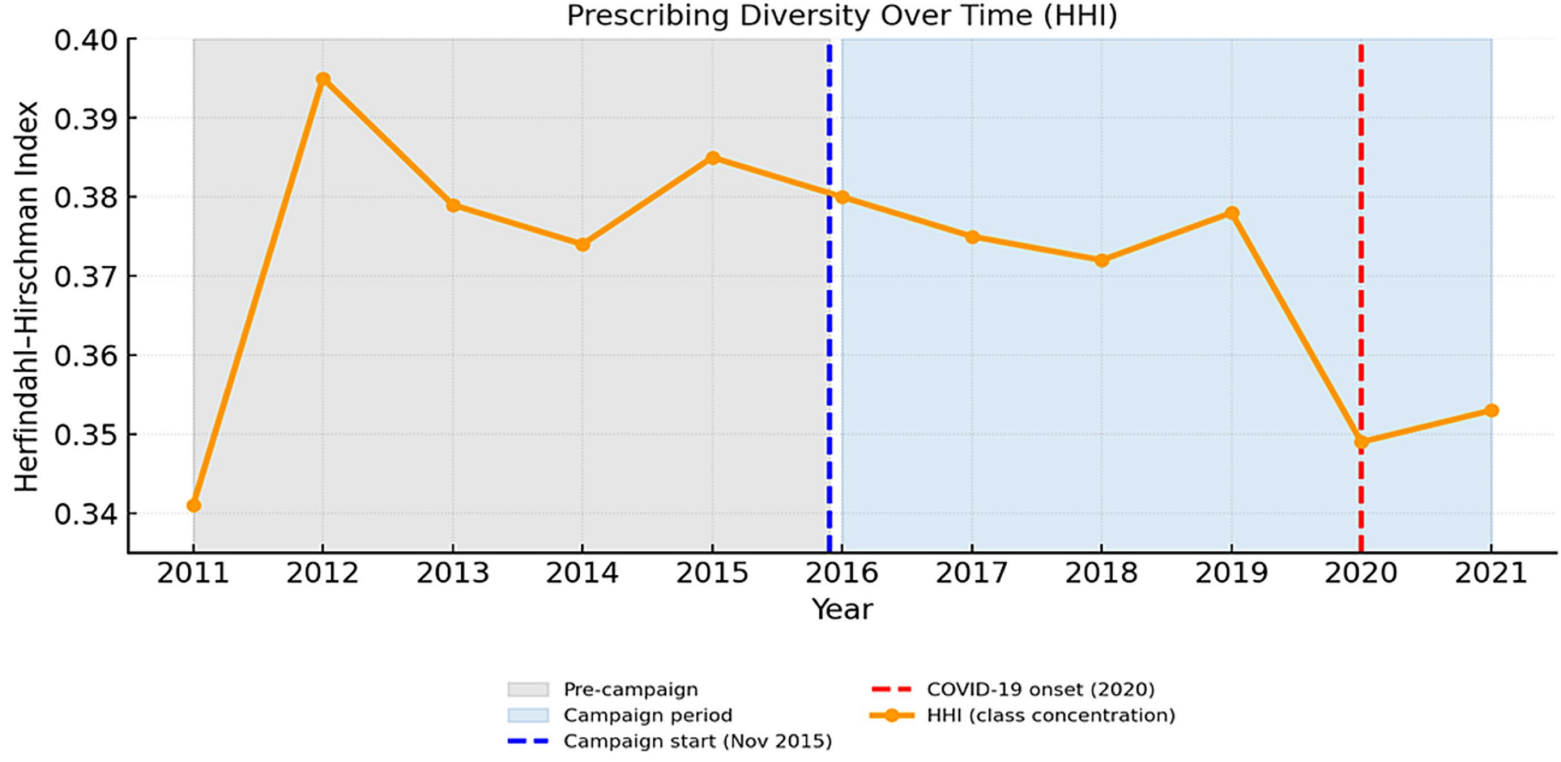
Trends in prescribing diversity measured by the Herfindahl–Hirschman index (HHI), 2011–2021. [Shaded areas denote pre-campaign (2011–2015) and campaign (2016–2021) periods; dashed lines mark the 2015 campaign onset (blue) and COVID-19 onset (red)].

Figure 7 illustrates yearly variations in prescribing diversity from 2011 to 2021, assessed using the Herfindahl–Hirschman Index (HHI). The index rose steadily from 0.309 in 2011 to approximately 0.340–0.346 by 2020–2021, indicating a gradual but meaningful increase in prescribing concentration. This pattern reflects reduced diversity in antibiotic class selection, with prescriptions increasingly dominated by penicillins, cephalosporins, and macrolides.

A notable shift occurred around 2020, coinciding with the onset of the COVID-19 pandemic, although overall concentration levels remained largely stable. These findings suggest that, despite the decline in total prescription volumes during the campaign period, the structural composition of antibiotic use remained focused on a few dominant classes rather than diversifying toward a wider spectrum of agents.

### 3.11 Changes in the composition of prescribed antibiotics (2016–2021)

**Figure 8:**
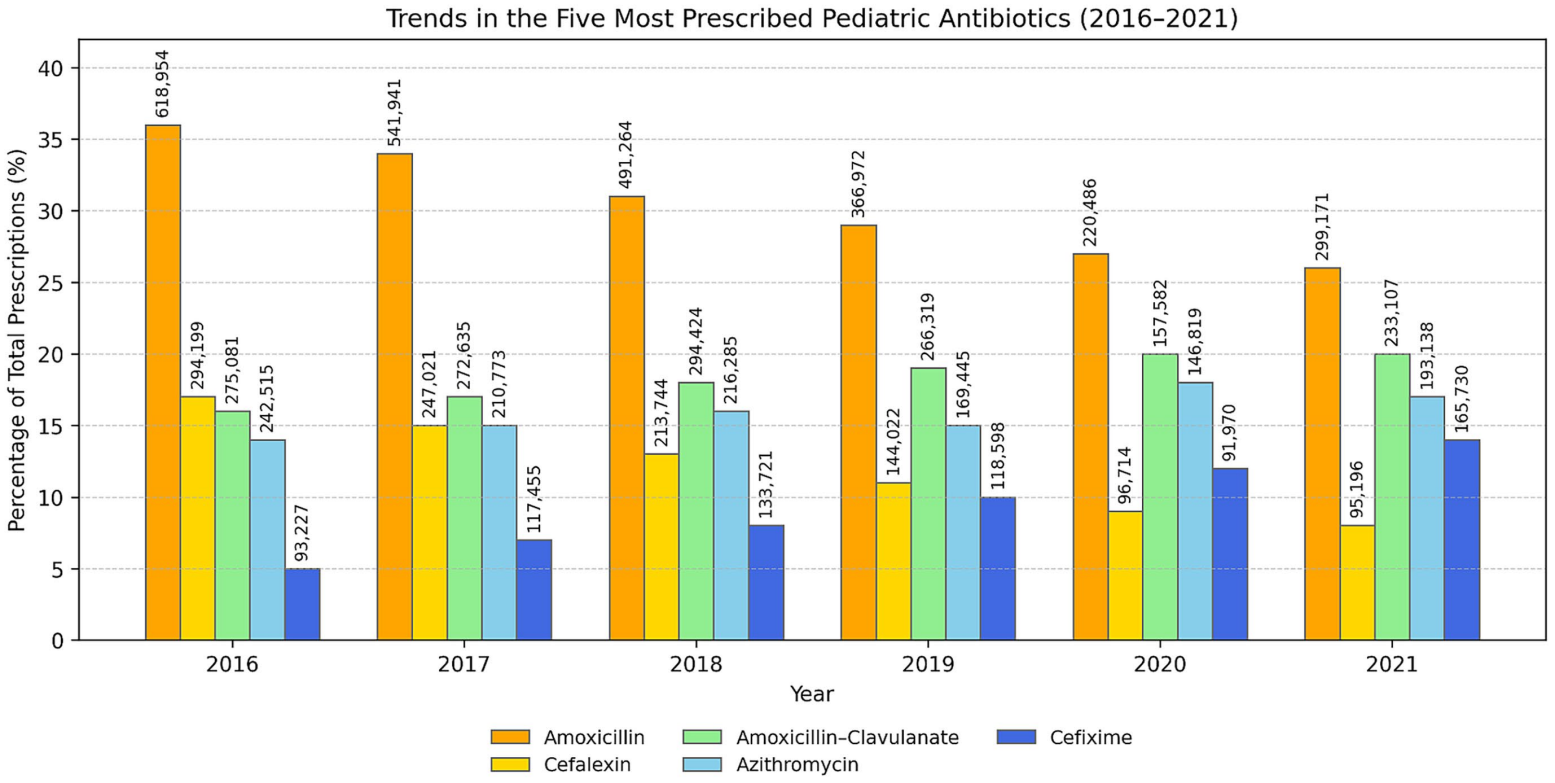
Proportional trends of the five most prescribed antibiotics (2016–2021). Numbers above bars indicate absolute prescription counts (*n*).

**Figure 9:**
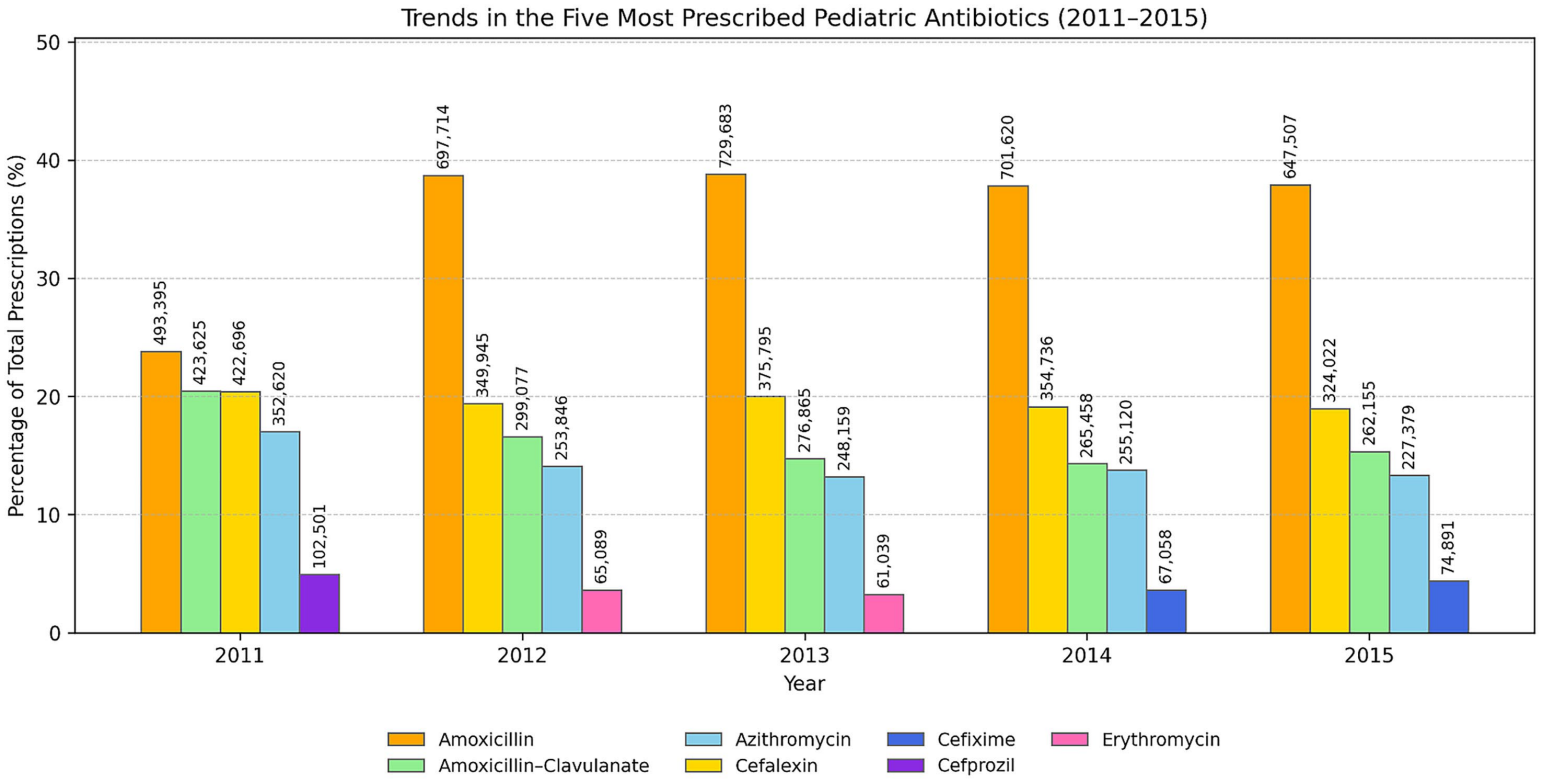
Proportional trends of the five most prescribed antibiotics (2011–2015). Numbers above bars indicate absolute prescription counts (*n*).

Figure 8 illustrates the proportional variations in the five most frequently prescribed antibiotics for pediatric patients from 2016 to 2021. Figure 9 presents the corresponding 2011–2015 data, with absolute prescription counts (*n*) annotated on each bar. The utilization of amoxicillin exhibited a consistent decline, decreasing from 36% in 2016 to 26% in 2021, which indicates a relative reduction of nearly one-third throughout the observed timeframe. A comparable trend was observed for cefalexin, which declined from 17% to 8%, indicating the overall decrease in the prescribing of first-generation cephalosporins.

Conversely, the prescriptions for amoxicillin-clavulanic acid increased steadily from 16 to 20%, reflecting an ongoing inclination toward broader-spectrum *β*-lactam combinations, even in light of stewardship initiatives. Azithromycin exhibited a consistent pattern, oscillating between 14 and 18%, with a notable peak in 2020, probably indicative of its short-term empirical application during the COVID-19 pandemic. Cefixime, a notable third-generation cephalosporin, rose from 5% in 2016 to 14% in 2021, indicating an increasing trend in the utilization of more potent oral cephalosporins.

In summary, although the overall rate of antibiotic prescribing has decreased, there has been a notable shift in the types of agents being utilized. Narrow-spectrum antibiotics like amoxicillin and cefalexin are increasingly being substituted with broader-spectrum options such as amoxicillin-clavulanate, azithromycin, and cefixime.

### 3.12 Amoxicillin index (2011–2021)

**Figure 10:**
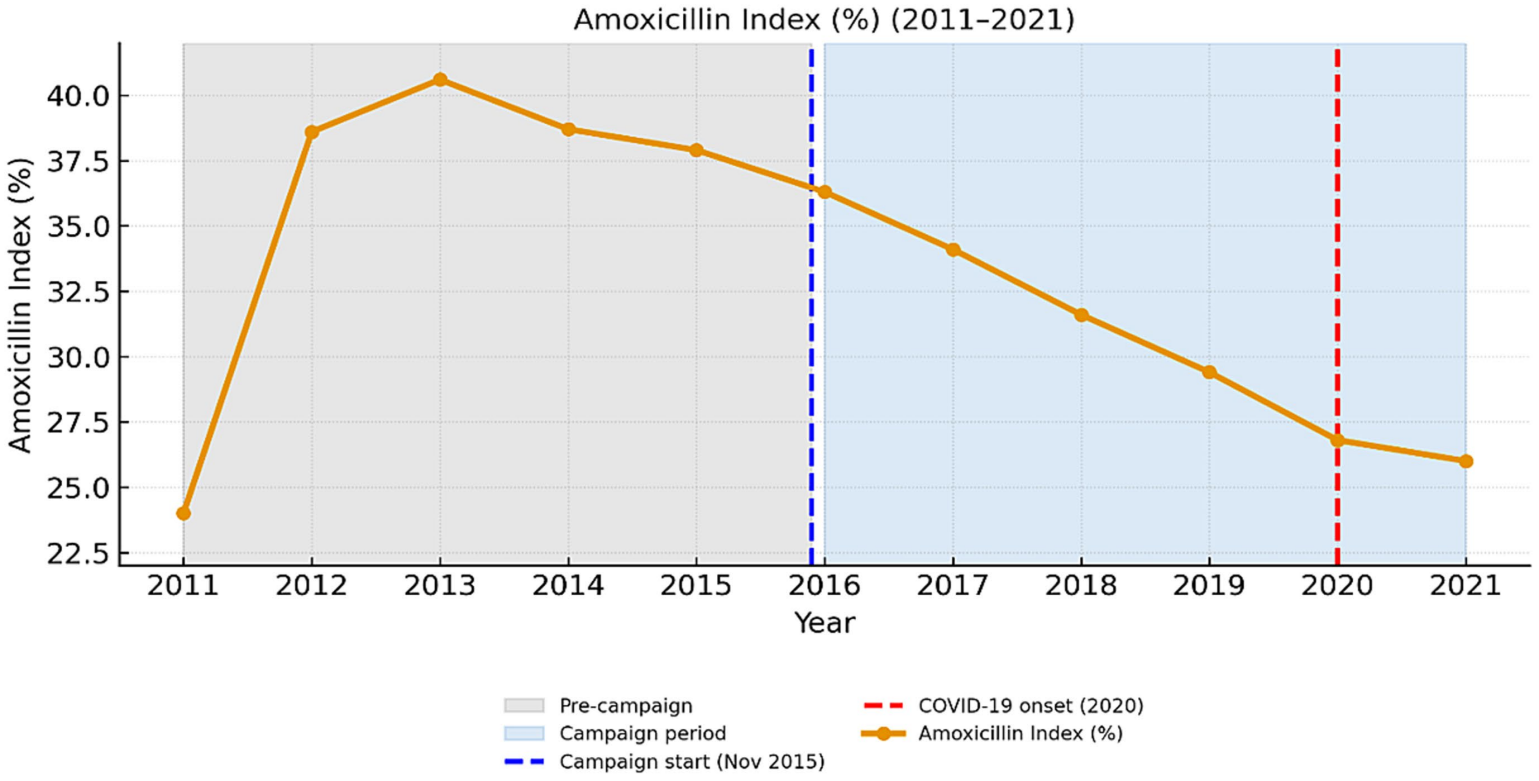
Amoxicillin index (%) in pediatric prescriptions from 2011 to 2021.

Figure 10 presents the temporal evolution of the *Amoxicillin Index* from 2011 to 2021, highlighting long-term changes in prescribing quality within the penicillin class. The index exhibited a biphasic pattern, showing a sharp increase from 23.9% in 2011 to a peak of 40.5% in 2013, followed by a steady decline that returned values close to baseline by 2021 (25.6%). Across the entire timeframe, the compound annual growth rate (CAGR) was modestly positive (+0.69% per year), whereas the linear regression slope indicated a negative trend (−0.82 percentage points per year; *β* = −0.46, *p* = 0.15, *R*^2^ = 0.22), confirming a non-linear pattern. From 2011 to 2019, the observed change was not statistically significant (−0.25 pp./year, *p* = 0.75). However, during the COVID-19 period (2020–2021), the decline steepened (−1.14 pp./year), indicating an accelerated reduction in first-line amoxicillin use. Despite the reduction in total antibiotic prescription volumes, the trajectory of the *Amoxicillin Index* suggests that prescribing quality remained inconsistent, with a gradual shift toward broader-spectrum agents (Figure 10).

Overall, the Amoxicillin Index (Figure 10) indicates that while the overall antibiotic usage in children has decreased over time, there has been a notable shift in the prescribing patterns toward broader-spectrum agents. This highlights the importance of ongoing education for prescribers and the necessity of strict compliance with clinical guidelines to encourage appropriate antibiotic selection and avert the continued increase in broad-spectrum usage.

### 3.13 WHO AWaRe classification of prescribed antibiotics (2011–2021)

**Figure 11:**
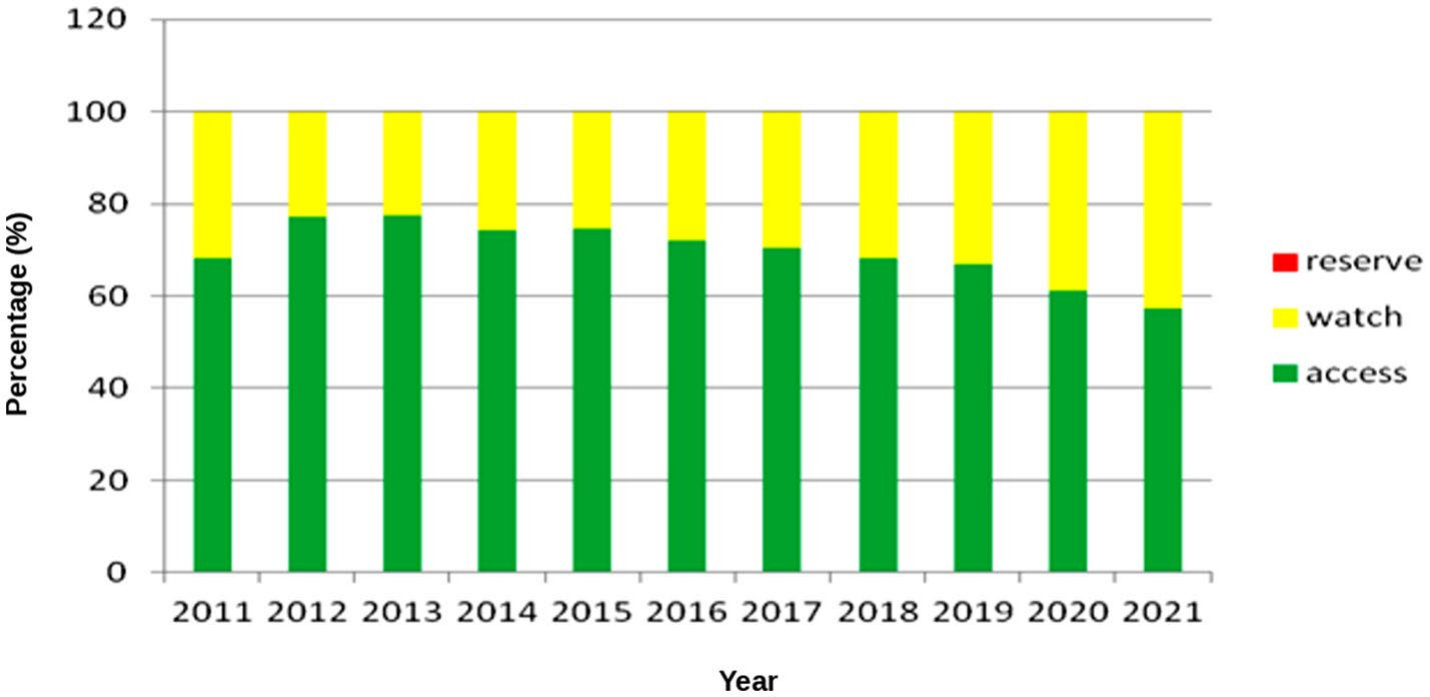
Distribution of antibiotic prescriptions (%) by WHO AWaRe classification, 2011–2021.

Throughout the study period, antibiotic use in the community pediatric sector predominantly adhered to WHO AWaRe recommendations (Figure 11). Between 2011 and 2020, agents from the *Access* group consistently represented more than 60% (Figure 7) of total prescriptions, fluctuating annually between approximately 61% and 77%. The proportion of *Watch* antibiotics remained stable with a slight upward trend, whereas *Reserve* agents were virtually absent throughout. In 2021, the share of *Access* antibiotics declined to around 58%, falling marginally below the 60% benchmark. This transient deviation coincided with the COVID-19 pandemic and was likely influenced by disruptions in healthcare access and temporary shifts in prescribing practices.

### 3.14 Trends in pediatric respiratory diagnoses (2016–2021)

**Figure 12:**
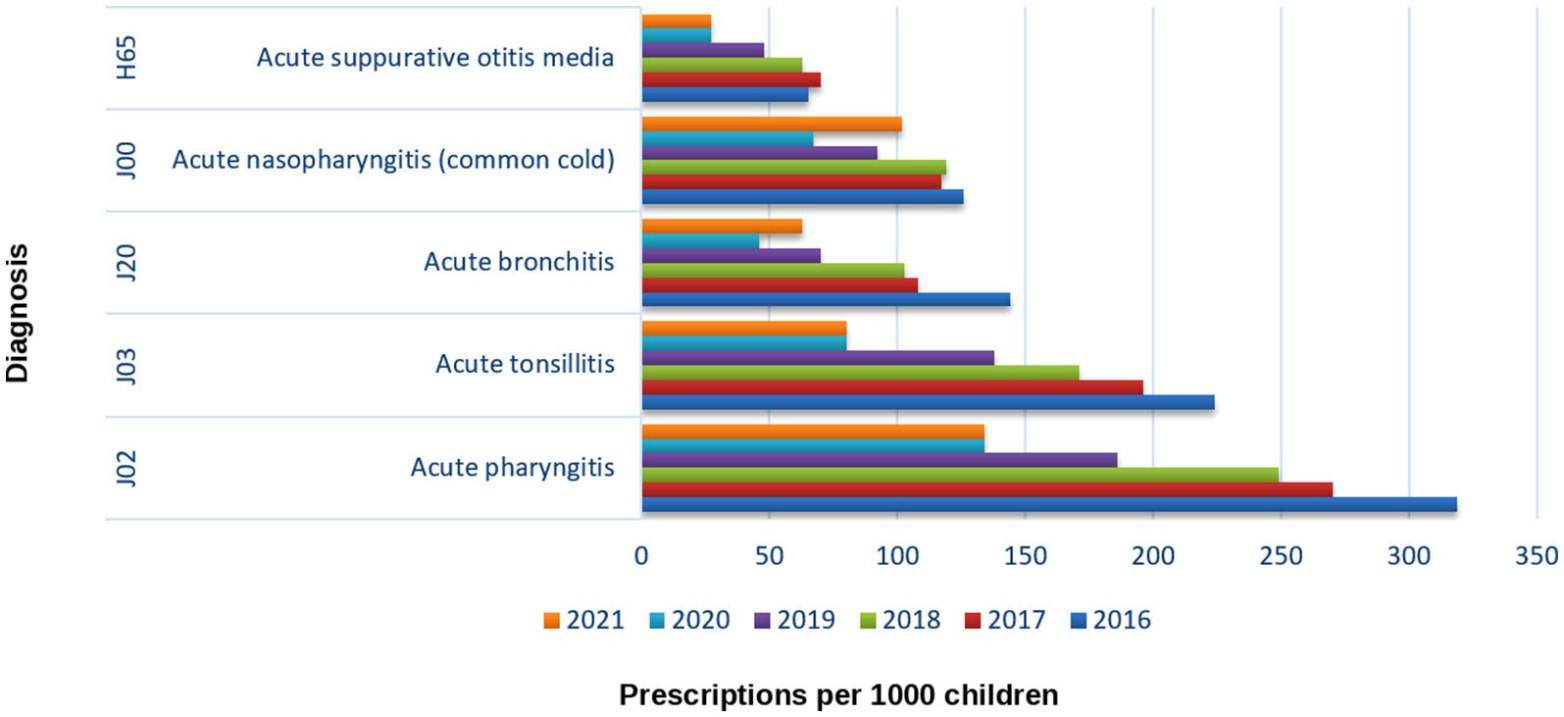
Rate of pediatric antibiotic prescriptions by diagnosis (2016–2021).

Figure 12 presents the annual counts of antibiotic prescription rates for the five predominant pediatric respiratory diagnoses acute pharyngitis (J02), acute tonsillitis (J03), acute bronchitis (J20), common cold (J00), and suppurative otitis media (H65) from 2016 to 2021. Over the course of 6 years, a notable and steady decrease was recorded for almost all conditions, particularly for pharyngitis and tonsillitis.

Statistical analysis through linear regression demonstrated significant decreases in prescribing practices for all major respiratory infections, with the exception of the common cold. In the case of acute pharyngitis (J02), there was an observed decrease in prescription rates averaging 39.9 per year (95% CI −51.0 to −28.8, *p* < 0.001, *R*^2^ = 0.96). Similarly, acute tonsillitis (J03) exhibited a notable decline of 31.5 per year (95% CI −39.9 to −23.1, *p* < 0.001, *R*^2^ = 0.96). Prescription rates for acute bronchitis (J20) decreased by 17.8 annually (95% CI −27.8 to −7.8, *p* = 0.008, *R*^2^ = 0.86), while those for suppurative otitis media (H65) declined by 9.5 per year (95% CI −14.9 to −4.2, *p* = 0.008, *R*^2^ = 0.86). The common cold (J00) showed a slight, non-significant decline of 8.5 prescriptions annually (95% CI −19.7 to +2.7, *p* = 0.10, *R*^2^ = 0.53), indicating ongoing empirical antibiotic usage for self-limiting viral infections.

From 2016 to 2021, there was a notable decrease in antibiotic prescription rates: acute pharyngitis saw a reduction of about 58% (from 319 to 134 prescriptions), acute tonsillitis declined by 64% (from 224 to 80), acute bronchitis decreased by 56% (from 144 to 63), and otitis media experienced a 58% decrease (from 65 to 27). The decrease for the common cold was somewhat less pronounced, approximately 19% (from 126 to 102). The observed patterns indicate the sustained impact of national educational initiatives aimed at encouraging rational antibiotic use among children.

An evident shift took place in 2020, aligning with the beginning of the COVID-19 pandemic. That year saw the lowest prescription counts for almost all respiratory diagnoses, a decrease that closely aligned with public health measures such as school closures, mask usage, and social distancing, which together diminished the spread of common respiratory pathogens. In 2021, there was a modest increase in prescription rates for bronchitis and the common cold (46 to 63 and 67 to 102, respectively), while pharyngitis, tonsillitis, and otitis media remained close to their historical lows.

The findings suggest that the national educational campaign led to a consistent, nearly linear decrease in antibiotic usage for pediatric respiratory infections, with the COVID-19 pandemic causing a temporary intensification of this trend. The partial rebound noted in 2021 probably signifies a reversion to pre-pandemic infection trends rather than a setback in stewardship advancements.

## 4 Discussion

The multisectoral, six-year-long Campaign for the Rational Use of Antibiotics implemented according to the ECDC methodology in Serbia led to 32.8% reduction in the number of prescribed systemic antibiotics per 1,000 children between 2015 and 2021. These results are in line with Spain’s PRAN program which documented an approximate 30% reduction in pediatric and community prescribing (7), and Lithuania’s observed comparable improvements through integrated educational and regulatory actions (9). In Germany, nationwide insurance data covering over 9 million children demonstrated a 43% decline in outpatient prescriptions between 2010 and 2018 (10). Italy recorded a 4% annual reduction from 2012 to 2018 (12) and Britain conducted, a system wide quality-improvement initiative combining the *TARGET* toolkit and *Healthier Together* program reduced antibiotic prescribing in children under five by 20%, without increasing healthcare visits (13). Collectively, these European experiences confirm that sustained, multifaceted interventions integrating education, feedback, and stewardship frameworks can substantially reduce inappropriate antibiotic use across pediatric primary care settings. Similar findings were noted in nationwide French campaign between 2002 and 2007 achieved a 26% decrease in outpatient antibiotic use, (11). The magnitude and persistence of Serbia’s decline, confirmed by segmented regression, position it within the top tier of European stewardship outcomes and confirms the effectiveness of sustained, multimodal interventions as documented in large-scale systematic reviews (3, 6, 10). The effect was gradual rather than abrupt, with the steepest decreases recorded during the active campaign years, confirming a cumulative behavioral change in prescribing practices. These findings extend earlier Serbian observations (26), and mirror long-term European experiences documented by earlier works and by systematic reviews emphasizing the superiority of sustained multimodal educational approaches (1, 3, 13, 27).

The national campaign, coordinated by the Serbian Ministry of Health was one of the most comprehensive professional and public antibiotic-stewardship initiatives ever conducted in Serbian pediatric primary care. It was financially supported through a World Bank loan, which allowed large-scale dissemination of educational materials, media communication, and the creation of digital infrastructure for antibiotic monitoring. Across 6 years, more than 8,700 brochures, 2,500 posters, and 3,000 additional educational resources were distributed to 158 primary-care centers, while more than 2,100 pediatricians and 6,000 pharmacists participated in training workshops and CME courses. The campaign coincided with the introduction of electronic prescriptions into the Integrated Health Information System (IHIS) in 2019, ensuring comprehensive digital capture of pediatric prescription antibiotic data (28).

The temporal dynamics confirmed a consistent and statistically significant decline in prescribing intensity. The maximum 61% reduction in systemic antibiotic prescriptions per 1,000 children observed between 2011 and 2020 before partial rebound in 2021 reflects the cumulative effect of the long-term national campaign initiated in November 2015, as well as the contribution of two earlier short-term initiatives. It is among the most pronounced decade-long decline reported in Central and Southeastern Europe and within the highest in the EU/EEA region (7, 9, 10, 12, 13). From 2011 to 2015, prescription rates per 1,000 children remained relatively stable, followed by a progressive reduction beginning late 2015 when the long term campaign launched. Interrupted time-series analysis demonstrated a significant post-intervention acceleration of the downward slope (*p* < 0.05), independent of the transient pandemic effect. Although a further sharp decrease occurred in 2020 due to COVID-19 (lockdowns, school closures, reduced respiratory-virus circulation), similar temporary contractions were reported across the EU/EEA and United States (8, 9, 20, 29). The partial rebound in 2021 did not reach pre-intervention levels, indicating that most of the behavioral gains achieved through the campaign persisted despite pandemic disruptions.

Age-stratified analyses showed the largest reductions in the two youngest cohorts (2–23 months and 2–11 years respectively), which also had the highest baseline prescribing rates. This pattern is consistent with the target audience of the educational activities, which were primarily directed toward preschool and early school-aged children and their parents, and more broadly toward parents and caregivers of younger children. Smaller but still measurable decreases occurred among neonates and adolescents. This uniform downward direction across all pediatric subgroups demonstrates that stewardship measures effectively reached both the most heavily treated and lower-exposure populations. The “convergent decline” pattern indicates that prescribers reduced use most in age groups historically associated with unnecessary antibiotic exposure, confirming that educational interventions successfully targeted high-impact segments of pediatric practice.

Across all antibiotic classes, prescribing rates per 1,000 children showed a consistent decline during the observation period, confirming that the reduction was not limited to a single antibiotic group. Penicillins remained the most prescribed group but exhibited a gradual downward trajectory, whereas cephalosporins and macrolides displayed more variable patterns. Within *β*-lactams, first- and second-generation cephalosporins decreased substantially, while third-generation agents increased in relative share, indicating a redistribution rather than a uniform contraction. This pattern parallels those observed in other European countries undergoing transitional stewardship phases, where total prescribing falls but prescribers shift toward broader agents they perceive as more reliable (9, 10, 12, 13).

The Herfindahl–Hirschman (HHI) index revealed a moderate rise in prescribing concentration, signifying that a smaller set of agents accounted for a growing proportion of total use. Such consolidation can partially reflect improved adherence to clinical guidelines fewer, guideline-preferred choices but excessive concentration carries the risk of selecting resistance within these dominant classes. In this context, the Serbian trajectory mirrors the ESAC quality-indicator experience, where moderate homogenization was interpreted as “partial rationalization” rather than optimal diversification (30).

Analysis of individual agents provides further granularity. Over the decade, the relative contribution of amoxicillin-clavulanate increased from 16% to 20%, cefixime from 5% to 14%, and azithromycin from 14% to 18%, while cephalexin declined from 17% to 8%. These changes, visualized in the antibiotic-specific histogram, illustrate that despite the substantial quantitative decrease in total prescriptions, prescribers still tend to “upgrade” coverage when uncertain about etiology, a behavioral trend repeatedly reported in Spain, Lithuania, and Singapore (7, 9, 31). The increase in third-generation cephalosporins and macrolides underscores the ongoing need for diagnostic support and electronic feedback mechanisms between primary-care pediatricians and infectious-disease specialists.

The Amoxicillin Index (AI) representing the proportion of amoxicillin and phenoxymethylpenicillin among all pediatric antibiotic prescriptions declined throughout the study period. The lower AI reflects both the gradual shift in prescribing toward broader-spectrum agents as well as limited market availability of phenoxymethylpenicillin after 2015 a problem shared by many European markets (9, 10, 12) Nevertheless, the overall Access-group share remained at or above 60% throughout most of the observation period and decreased marginally to 58% only in 2021, a temporary deviation from the WHO benchmark of ≥60% set in the 6th Model List of Essential Medicines for Children (EMLc) (32). This brief decline underscores the need to sustain supply chains for first-line agents to maintain long-term AWaRe compliance.

Diagnosis-specific analyses further corroborate this interpretation. The largest absolute decreases were registered for pharyngitis, tonsillitis, otitis media, and bronchitis, conditions that together accounted for most inappropriate antibiotic use before the intervention. By contrast, prescribing for the *common cold* a predominantly viral condition showed only minor change, indicating that although diagnostic precision improved for bacterial-suspect infections, residual inappropriate use for viral illnesses persists. This diagnostic selectivity mirrors findings from pediatric stewardship programs in Spain, Lithuania, and Singapore, where public and professional campaigns improved adherence to clinical guidelines for respiratory tract infections (7, 9, 31). The observed shift aligns with the European Surveillance of Antimicrobial Consumption (ESAC) framework, which designates diagnostic specificity and reduced use for viral illnesses as core quality indicators (30). The above indications, had the majority emphasis of the media and educational intervention.

Overall, these findings confirm that the Serbian campaign achieved meaningful rationalization both quantitative and qualitative, yet underline the need for continued focus on differential diagnosis and class optimization in future stewardship efforts.

Further evidence of increased public understanding aligns with external survey data. According to the *Health Report 2020,* an independent survey conducted by the Stada Group (33), 54% of Serbian respondents demonstrated knowledge of the appropriate use of antibiotics, one of the highest proportions in Europe and comparable to Austria (53%). However, only 61% of respondents reported concern about antibiotic-resistant bacteria, slightly below the European average of 66%, suggesting that while awareness of correct antibiotic use has improved, risk perception regarding antimicrobial resistance still requires reinforcement.

The campaign also aligns closely with the global stewardship priorities established by the WHO Global Action Plan on Antimicrobial Resistance (GAP-AMR) and the AWaRe framework. Maintaining Access-group predominance throughout most of the observation period, even amid pandemic disruptions, represents significant progress toward the WHO target of ≥60% for first-line agent (21). Integration into Antimicrobial Medicines Consumption (AMC) WHO has further enhanced feedback loops and transparency in national reporting, ensuring that Serbia’s wholesale antibiotic-consumption data contribute to regional AMR monitoring efforts (26). In addition, this 10-year overview of antibiotic prescriptions habits gives valuable insight into detailed consumption patterns in pediatric primary care. These developments mark a transition from initial awareness rising toward data-driven accountability, consistent with the evolving concept of stewardship maturity across Europe.

The COVID-19 pandemic introduced a temporary distortion in antibiotic-use dynamics. In 2020, community antibiotic prescribing in many EU/EEA countries declined by 25%–40%, largely because of reduced transmission of respiratory pathogens and limited healthcare access (5, 9, 20, 28). Serbia exhibited the same sharp contraction (−33.7%), followed by a partial rebound in 2021 (+41.7%). Importantly, the post-pandemic resurgence did not restore pre-intervention levels, indicating that the behavioral changes instilled through the campaign persisted beyond the crisis. Nevertheless, the transient overuse of macrolides driven by misconceptions about their antiviral efficacy underscored the fragility of stewardship gains under the pressure of misinformation (5, 9). This experience highlights the necessity of embedding stewardship principles into professional curricula and electronic prescribing systems to ensure resilience during future disruptions.

Sustaining the progress achieved through continuous education and digital oversight remains the central public-health priority. Longitudinal studies have shown that awareness-driven declines can reverse within three to 5 years after campaign visibility decreases, as observed in France (11). Incorporating stewardship modules into medical and pharmacy education, expanding diagnostic support, and implementing audit-and-feedback functions within Serbia’s IHIS e-prescription platform are critical to preserving long-term gains. As recent expert reviews emphasize, countries must shift from asking “how many antibiotics are used” to “which antibiotics are used,” focusing on qualitative optimization rather than volume alone (34).

In summary, a decade of national surveillance confirms that Serbia’s multisectoral stewardship campaign led to one of the largest and most sustained reductions in pediatric antibiotic use in Europe. The results demonstrate that sustained public and professional engagement, underpinned by continuous monitoring and education, can yield durable behavioral change. Future priorities should focus on restoring Access-class antibiotic proportions above 70%, reducing the reliance on macrolides and third-generation cephalosporins, and linking consumption data with resistance trends through the GLASS system. Serbia’s experience exemplifies the effectiveness of coordinated national action in promoting rational pediatric antibiotic use and provides a replicable model for stewardship implementation in alignment with WHO and ECDC objectives.

## Data Availability

The datasets presented in this article are not readily available because the original dataset is confidential information provided by the Serbian Ministry of Health. Requests to access the datasets should be directed to Serbian Ministry of Health, kabinet@zdravlje.gov.rs.
