# Supplementary material for: Long-term public antibiotic awareness campaign significantly reduced inappropriate antibiotic use in pediatric primary care settings
Source: Front Public Health. 2026 Feb 9;14:1730266. doi: 10.3389/fpubh.2026.1730266 (PMC12928503; doi:10.3389/fpubh.2026.1730266)
Supplement: Supplementary file 1 [file Data_Sheet_1.pdf]

Република Србија
МИНИСТАРСТВО ЗДРАВЉА

ДРУГИ ПРОЈЕКАТ
РАЗВОЈА
ЗДРАВСТВА
СРБИЈЕ

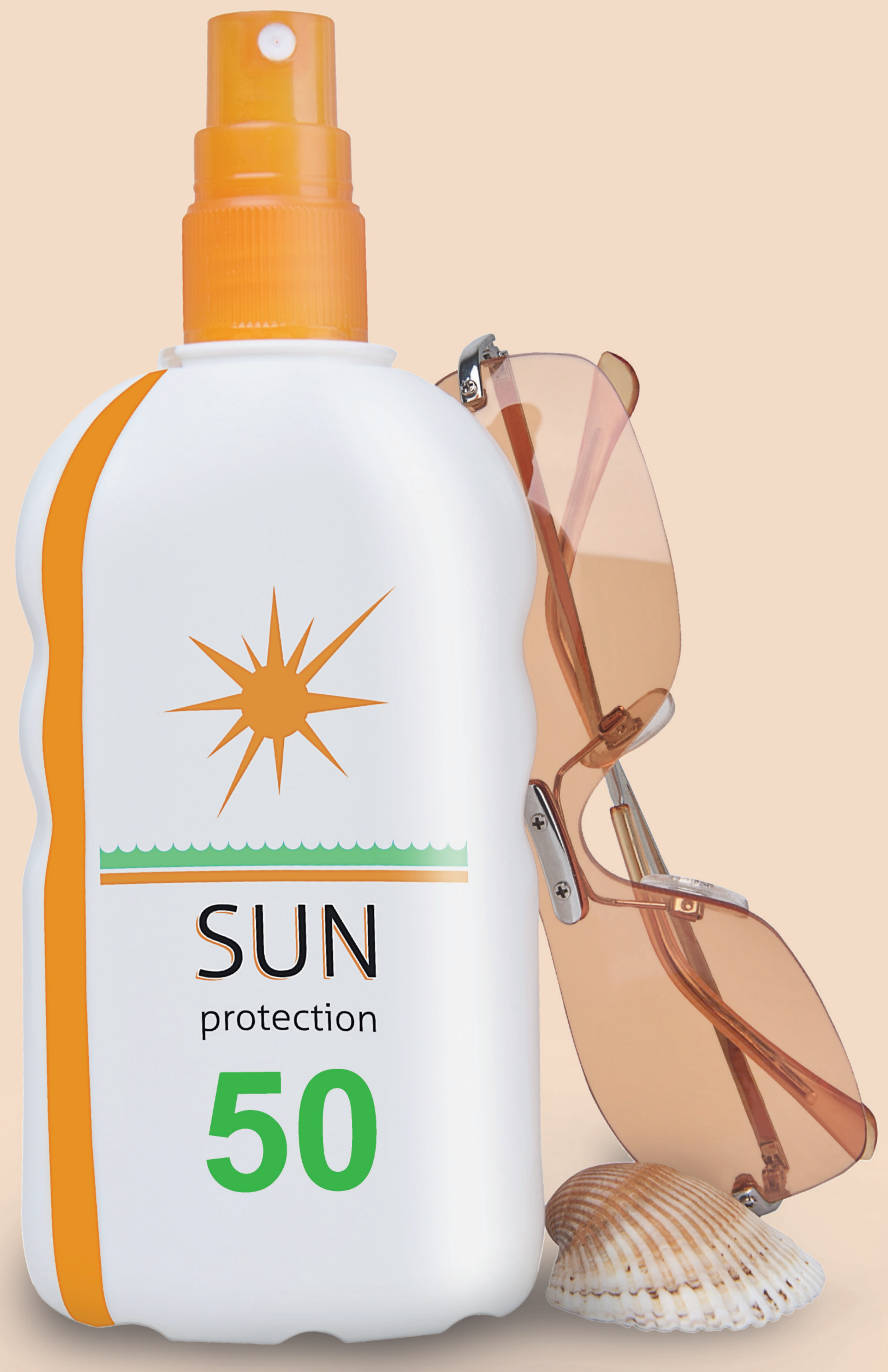

Да ли је ово добра заштита од прехладе или грипа?

Нису ни антибиотици.

© Thinkstock

Антибиотици.

Користите их паметно -
никад против
прехладе и грипа.

EUROPEAN
ANTIBIOTIC
AWARENESS DAY

A EUROPEAN
HEALTH INITIATIVE

ИНСТИТУТ ЗА ЈАВНО ЗДРАВЉЕ СРБИЈЕ „Др Милан Јовановић Батут“
alims
УНИВЕРЗИТЕТ У БЕОГРАДУ МЕДИЦИНСКИ ФАКУЛТЕТ
СРПСКО ЛЕКАРСКО ДРУШТВО
SFUS Savez farmaceutskih udruženja Srbije
РЕПУБЛИКА СРБИЈА ФАРМАЦЕУТСКА КОМОРА
КОМОРА БИОХЕМИЧАРА СРБИЈЕ
STOMATOLOŠKA KOMORA SRBIJE
SUAH
УДРУЖЕЊЕ ПЕДИЈАТАРА СРБИЈЕ
UDRUŽENJE INTENZIVISTA SRBIJE
SPAS
gnezdo
RODITELJ
