# Supplementary material for: Long-term public antibiotic awareness campaign significantly reduced inappropriate antibiotic use in pediatric primary care settings
Source: Front Public Health. 2026 Feb 9;14:1730266. doi: 10.3389/fpubh.2026.1730266 (PMC12928503; doi:10.3389/fpubh.2026.1730266)
Supplement: Supplementary file 2 [file Data_Sheet_2.pdf]

Република Србија
МИНИСТАРСТВО ЗДРАВЉА

ДРУГИ ПРОЈЕКАТ
РАЗВОЈА
ЗДРАВСТВА
СРБИЈЕ

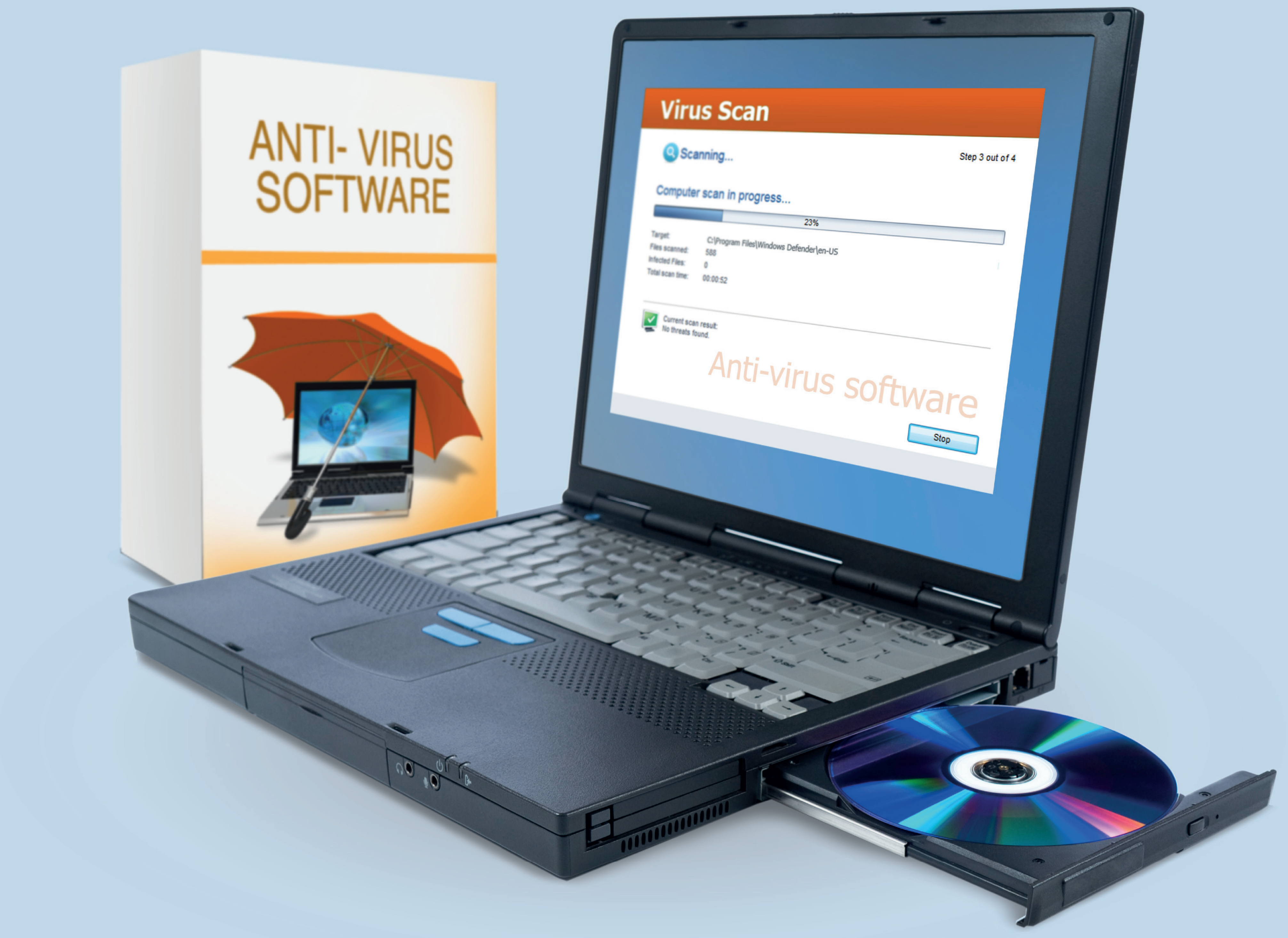

Да ли је ово добра заштита од прехладе или грипа?

Нису ни антибиотици.

© Thinkstock

Антибиотици.

Користите их паметно -
никад против
прехладе и грипа.

EUROPEAN
ANTIBIOTIC
AWARENESS DAY
A EUROPEAN
HEALTH INITIATIVE

ИНСТИТУТ ЗА ЈАВНО ЗДРАВЉЕ СРБИЈЕ „Др Милан Јовановић Батут"

alims

УНИВЕРЗИТЕТ У БЕОГРАДУ МЕДИЦИНСКИ ФАКУЛТЕТ

СРПСКО ЛЕКАРСКО ДРУШТВО

SFUS
Savez farmaceutskih udruženja Srbije

РЕПУБЛИКА СРБИЈА ФАРМАЦЕУТСКА КОМОРА
The Pharmaceutical Chamber of Serbia

КОМОРА БИОХЕМИЧАРА СРБИЈЕ

STOMATOLOŠKA KOMORA SRBIJE

SUAH

УДРУЖЕЊЕ ПЕДИЈАТАРА СРБИЈЕ

SPAS

gnezdo

RODITELJ
