# Supplementary material for: Long-term public antibiotic awareness campaign significantly reduced inappropriate antibiotic use in pediatric primary care settings
Source: Front Public Health. 2026 Feb 9;14:1730266. doi: 10.3389/fpubh.2026.1730266 (PMC12928503; doi:10.3389/fpubh.2026.1730266)
Supplement: Supplementary file 3 [file Data_Sheet_3.pdf]

Република Србија
МИНИСТАРСТВО ЗДРАВЉА

ДРУГИ ПРОЈЕКАТ
РАЗВОЈА
ЗДРАВСТВА
СРБИЈЕ

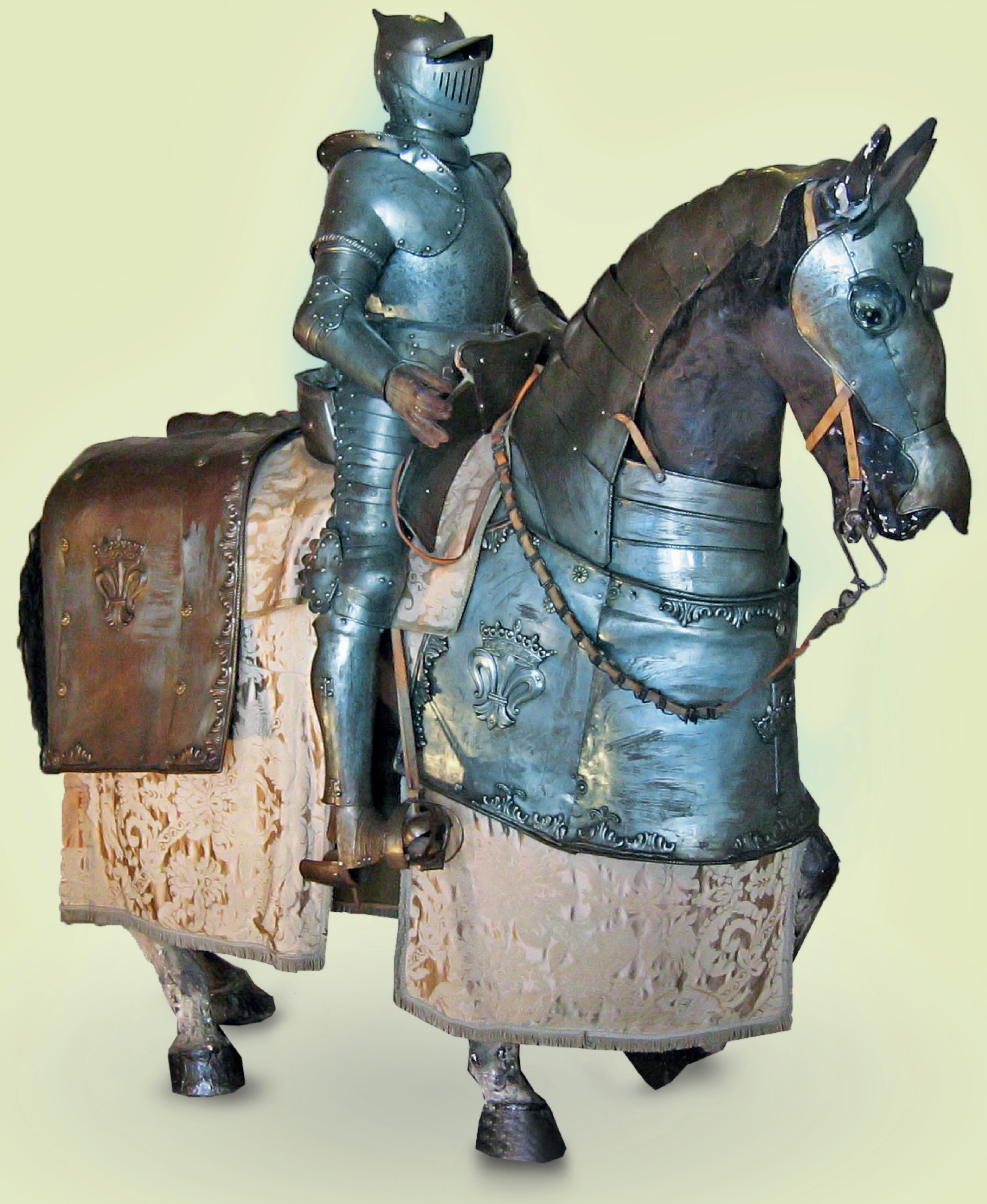

Да ли је ово добра заштита од прехладе или грипа?

Нису ни антибиотици.

© M.Stallbaum

Антибиотици.

Користите их паметно -
никад против
прехладе и грипа.

EUROPEAN
ANTIBIOTIC
AWARENESS DAY

A EUROPEAN
HEALTH INITIATIVE

ИНСТИТУТ ЗА ЈАВНО ЗДРАВЉЕ СРБИЈЕ „Др Милан Јовановић Батут“ · alims · УНИВЕРЗИТЕТ У БЕОГРАДУ МЕДИЦИНСКИ ФАКУЛТЕТ · СРПСКО ЛЕКАРСКО ДРУШТВО · SFUS Savez farmaceutskih udruženja Srbije · РЕПУБЛИКА СРБИЈА ФАРМАЦЕУТСКА КОМОРА The Pharmaceutical Chamber of Serbia · КОМОРА БИОХЕМИЧАРА СРБИЈЕ

STOMATOLOŠKA KOMORA SRBIJE · SUAH · УДРУЖЕЊЕ ПЕДИЈАТАРА СРБИЈЕ · UDRUŽENJE INTENZIVISTA SRBIJE · SPAS · gnezdo · RODITELJI
