# Supplementary material for: Long-term public antibiotic awareness campaign significantly reduced inappropriate antibiotic use in pediatric primary care settings
Source: Front Public Health. 2026 Feb 9;14:1730266. doi: 10.3389/fpubh.2026.1730266 (PMC12928503; doi:10.3389/fpubh.2026.1730266)
Supplement: Supplementary file 4 [file Data_Sheet_4.pdf]

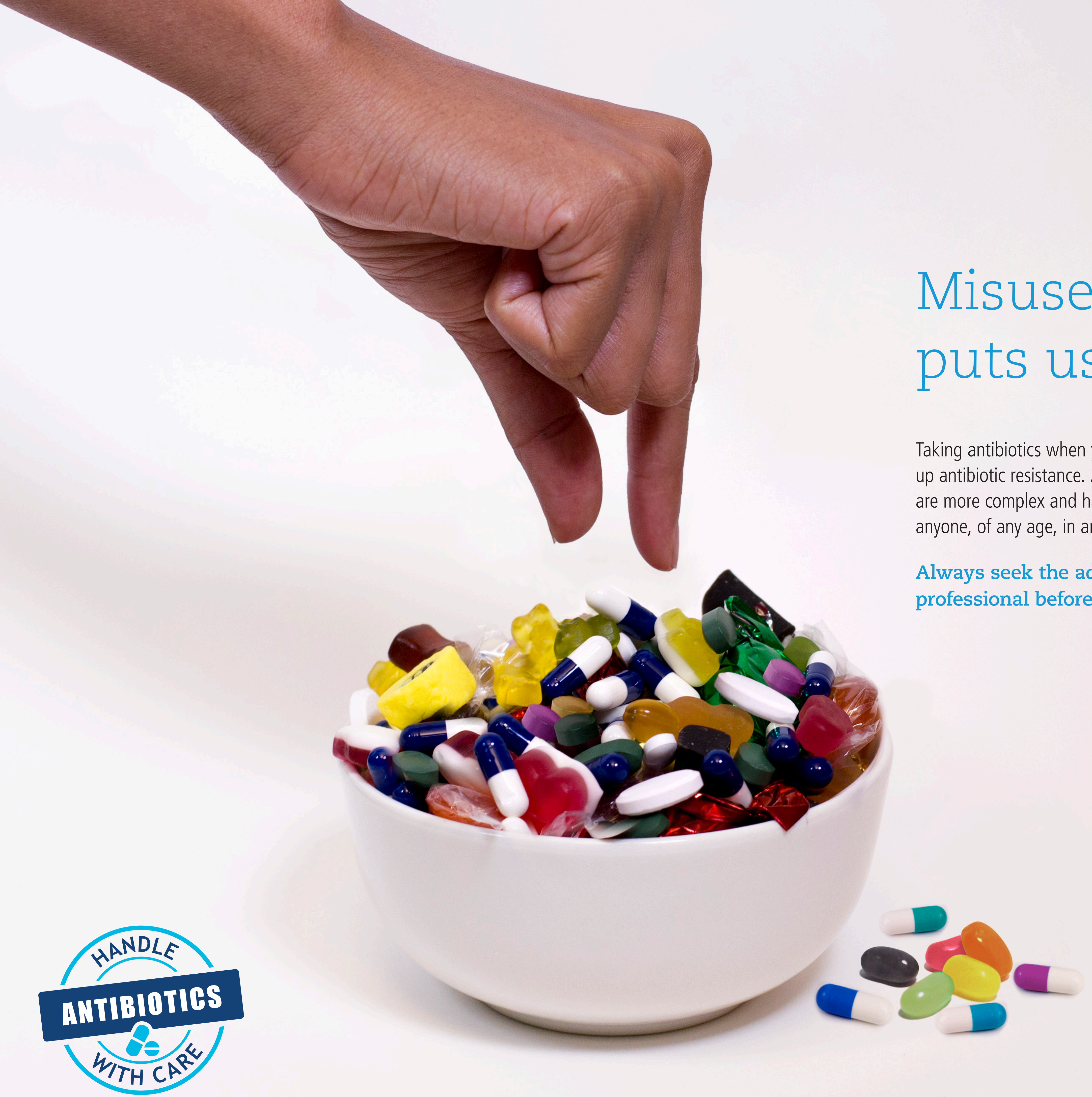

Misuse of **ANTIBIOTICS** puts us all at risk.

Taking antibiotics when you don't need them speeds up antibiotic resistance. Antibiotic resistant infections are more complex and harder to treat. They can affect anyone, of any age, in any country.

Always seek the advice of a healthcare professional before taking antibiotics.

HANDLE ANTIBIOTICS WITH CARE

World Health Organization
