# Supplementary material for: Long-term public antibiotic awareness campaign significantly reduced inappropriate antibiotic use in pediatric primary care settings
Source: Front Public Health. 2026 Feb 9;14:1730266. doi: 10.3389/fpubh.2026.1730266 (PMC12928503; doi:10.3389/fpubh.2026.1730266)
Supplement: Supplementary file 5 [file Data_Sheet_5.pdf]

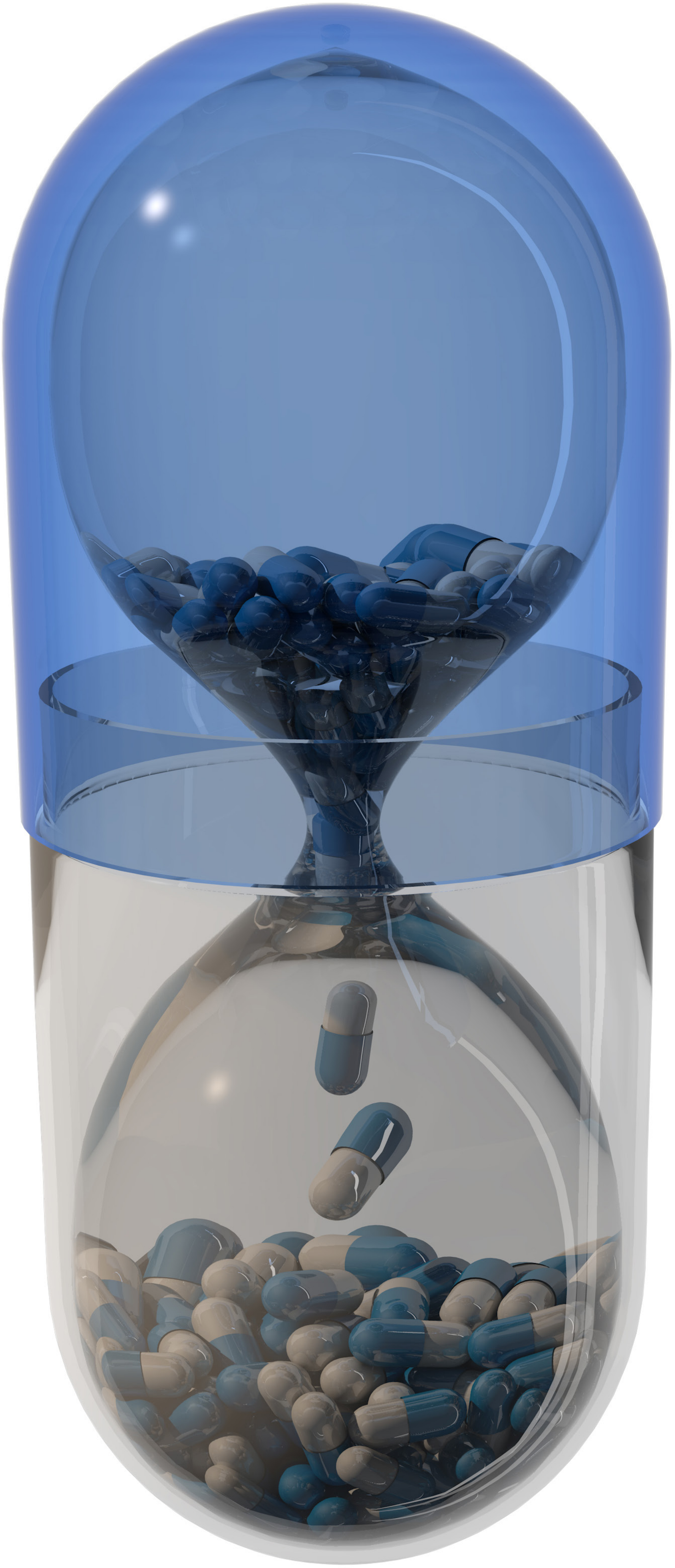

Our time with **ANTIBIOTICS** is running out.

Antibiotics are in danger of losing their effectiveness due to misuse and overuse, and in many cases they aren't even needed.

Always seek the advice of a healthcare professional before taking antibiotics.

HANDLE ANTIBIOTICS WITH CARE

World Health Organization
