# Supplementary material for: Long-term public antibiotic awareness campaign significantly reduced inappropriate antibiotic use in pediatric primary care settings
Source: Front Public Health. 2026 Feb 9;14:1730266. doi: 10.3389/fpubh.2026.1730266 (PMC12928503; doi:10.3389/fpubh.2026.1730266)
Supplement: Supplementary file 6 [file Data_Sheet_6.pdf]

Република Србија
МИНИСТАРСТВО ЗДРАВЉА

Кампања за рационалну употребу антибиотика

РАЗВОЈА
ЗДРАВСТВА
СРБИЈЕ

Растућа отпорност на антибиотике последње линије одбране

Карбапенеми су важна класа антибиотика последње линије одбране којима се лече бактеријске инфекције

Ширење инфекција отпорних на карбапенеме представља претњу за здравствену заштиту и безбедност пацијената у Европи јер озбиљно смањује могућност лечења инфекција

Сваке године, земље ЕУ/ЕЕП пријављују податке о отпорности на антибиотике Европској мрежи за надзор антимикробне отпорности (European Antimicrobial Resistance Surveillance Network EARS-Net), а о потрошњи антибиотика Европској мрежи за надзор потрошње антибиотика (European Surveillance of Antimicrobial Consumption Network ESAC-Net). Обе мреже раде у оквиру ECDC-а. По први пут, 18 земаља је EARS-Net-у пријавило податке за Acinetobacter spp. Осим тога, стручњаци из 38 европских земаља учествовали су у Европском истраживању ентеробактерија које производе карбапенемазу које је ECDC спровео на Универзитетском медицинском центру у Гронингену, у Холандији.

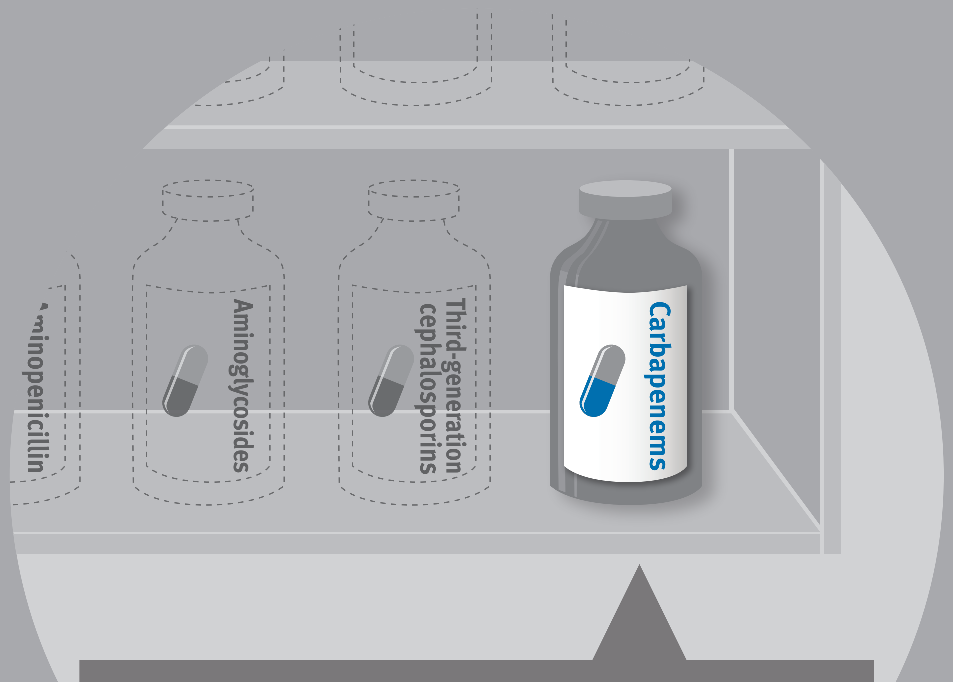

Карбапенеми су један од последњих избора које лекари имају када је реч о антибиотицима за лечење инфекција због бактерија које су отпорне на више антибиотика

Ентеробактерије отпорне на карбапенеме (ЕОК)

25/38 земаља пријавило је **одређену** епидемиолошку фазу

19/25

76% ових земаља поседује смернице о мерама за контролу инфекција за ЕОК

13/38 земаља пријавило је **неодређену** епидемиолошку фазу

2/13

15% ових земаља поседује смернице о мерама за контролу инфекција за ЕОК

Acinetobacter baumannii који производи карбапенемазу (АбПК)

2/38

5% свих земаља има смернице о мерама за контролу инфекција за АбПК

Многе европске земље немају смернице о превенцији и контроли инфекција отпорних на карбапенеме.

Мере за контролу ифекција

обазриво коришћење антибиотика

медицинска нега групе уз посебно опредељен, посвећен тим и опрему

мере хигијенске предострожности

вршите активан надзор и изолујте пацијента
