# Supplementary material for: Long-term public antibiotic awareness campaign significantly reduced inappropriate antibiotic use in pediatric primary care settings
Source: Front Public Health. 2026 Feb 9;14:1730266. doi: 10.3389/fpubh.2026.1730266 (PMC12928503; doi:10.3389/fpubh.2026.1730266)
Supplement: Supplementary file 7 [file Data_Sheet_7.pdf]

Република Србија
МИНИСТАРСТВО ЗДРАВЉА

Кампања за рационалну употребу антибиотика

ДРУГИ ПРОЈЕКАТ
РАЗВОЈА
ЗДРАВСТВА
СРБИЈЕ

Антибиотици
Будите одговорни

Сваке године, земље ЕУ/ЕЕП пријављују податке о отпорности на антибиотике Европској мрежи за надзор антимикробне отпорности European Antimicrobial Resistance Surveillance Network EARS-Net), а о потрошњи антибиотика Европској мрежи за надзор потрошње антибиотика (European Surveillance of Antimicrobial Consumption Network ESAC-Net). Обе мреже раде у оквиру ECDC-а.

Избијање и ширење отпорности на антибиотике, другим речима, способност бактерија да се одупру дејству антибиотика, постало је признат глобални проблем. Отпорност на антибиотике озбиљно ограничава број антибиотика који су доступни за терапију болести

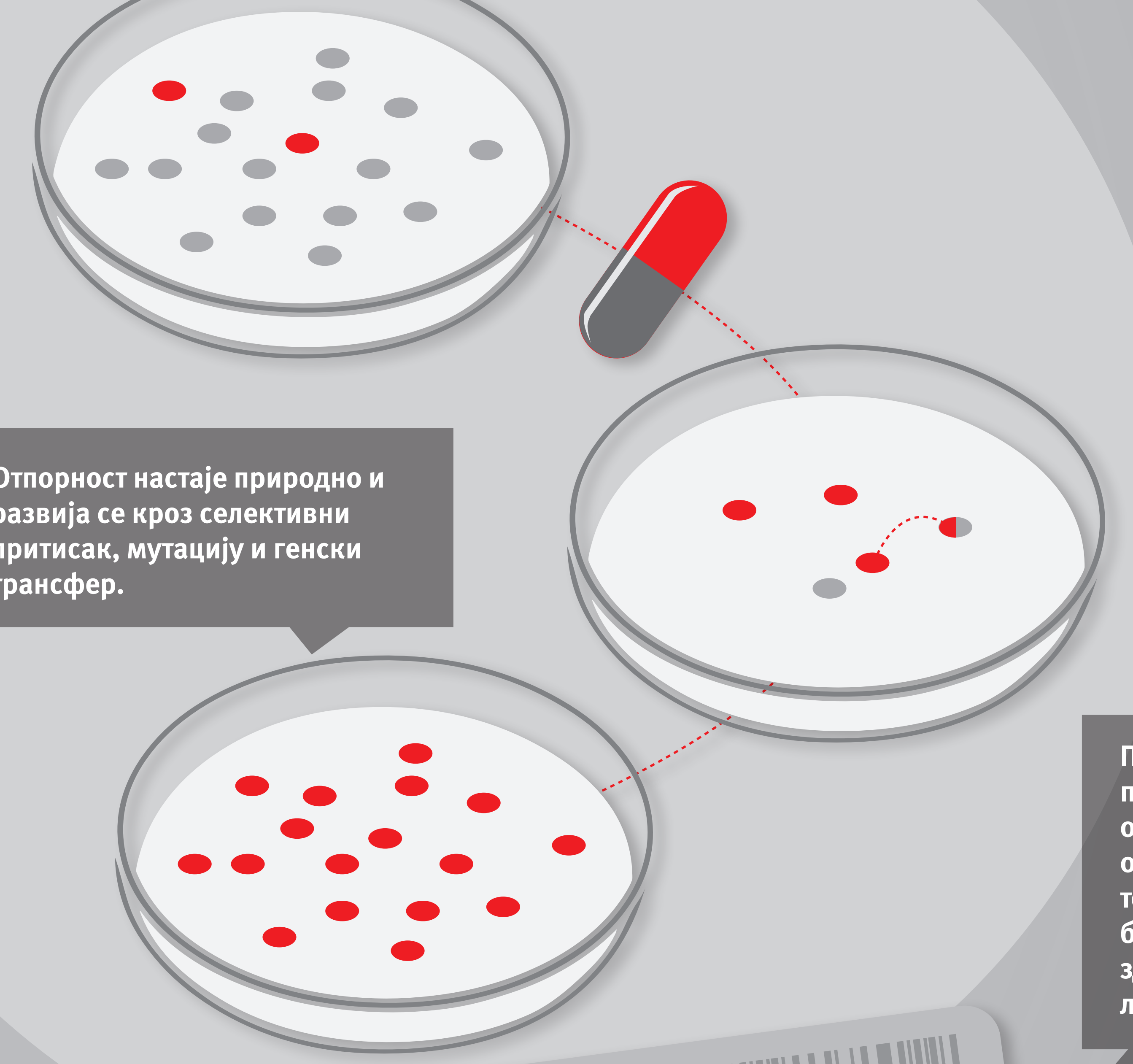

Отпорност настаје природно и развија се кроз селективни притисак, мутацију и генски трансфер.

Отпорност на антибиотике

Сваке године, у ЕУ око 25.000 пацијената умре од инфекција изазваних овим бактеријама отпорним на лекове.

Последице за болничке пацијенте обухватају одложено давање одговарајуће антибиотске терапије, дужи боравак у болници, веће трошкове здравствене заштите и лоше исходе по пацијенте.

Инфекције због ових вишеструко отпорних бактерија у ЕУ за последицу сваке године имају додатне здравствене трошкове у износу од најмање:

€ 1 500 000 000

Макролиди — *Streptococcus pneumoniae* — 8.7% Отпорност

Метицилин — *Staphylococcus aureus* — 17.8% Отпорност

Флуоро-квинолони — *Pseudomonas aeruginosa* — 21% Отпорност

Цефалоспорини треће генерације — *Klebsiella pneumoniae* — 25.7% Отпорност

Аминогликозиди високог нивоа — *Enterococcus faecalis* — 26.5% Отпорност

Аминопеницилини — *Escherichia coli* — 57.4% Отпорност
